# Computational Investigation of the Potential Antileishmanial Mechanism of the Nitroindazole Derivative VATR131

**DOI:** 10.3390/ph18101489

**Published:** 2025-10-03

**Authors:** Omar Casanova-Alvarez, Niurka Mollineda-Diogo, Aliuska Morales-Helguera, Vicente Arán-Redó, Reinaldo Molina-Ruiz, Norberto Sánchez-Cruz, Yendrek Velásquez-López, Yunierkis Perez-Castillo

**Affiliations:** 1 Computational Chemogenomics Group, Department of Physical Chemistry, Merida Research Unit, Institute of Chemistry, National Autonomous University of Mexico (UNAM), Mérida 97302, Mexico; ocasanova1997@gmail.com (O.C.-A.); norberto.sanchez@iquimica.unam.mx (N.S.-C.); 2 Centro de Bioactivos Químicos, Universidad Central “Marta Abreu” de Las Villas, Santa Clara 54800, Cuba; niurkam@uclv.cu (N.M.-D.); aliuska.morales@gmail.com (A.M.-H.); reymolina@uclv.edu.cu (R.M.-R.); 3 Instituto de Química Médica del Consejo Superior de Investigaciones Científicas de España, Juan de la Cierva 3, 28006 Madrid, Spain; uvejotaran@gmail.com; 4 Departamento de Procesos Químicos, Alimentos y Biotecnología, Facultad de Ingeniería y Ciencias Aplicadas, Universidad Técnica de Manabí, Av. Urbina y Che Guevara, Portoviejo 130104, Ecuador; yendrek_97@hotmail.com; 5 Grupo de Bio-Quimioinformática and Facultad de Ingeniería y Ciencias Aplicadas, Universidad de Las Américas, Quito 170125, Ecuador

**Keywords:** leishmanicidal activity, molecular docking, molecular dynamics, neglected tropical disease, nitroindazole

## Abstract

**Background:** Neglected tropical diseases (NTDs) significantly impact global health, particularly affecting impoverished communities. Among these diseases, leishmaniasis, caused by protozoan parasites of the genus *Leishmania* and transmitted through sandfly vectors, remains a challenge due to limited therapeutic options. Current treatments often suffer from significant limitations, such as high toxicity, limited efficacy, and the emergence of drug resistance. **Objectives:** This study investigates the potential antileishmanial mechanism of action of nitroindazole derivatives, specifically evaluating the compound VATR131, a molecule with notable selectivity and potency against *Leishmania infantum*. **Methods:** We employed computational methodologies, including target fishing, molecular docking, and atomistic molecular dynamics simulations, to identify and characterize potential molecular targets of VATR131. **Results:** The analysis revealed cysteine peptidase A as a promising target potentially mediating the antileishmanial activity of VATR131. Molecular dynamics simulations suggest critical hydrophobic interactions and hydrogen bonds between the compound and its most likely receptor, thus offering deeper insights into its potential mechanism of action. **Conclusions:** These findings contribute to the development of novel and effective therapies for leishmaniasis, highlighting the need for experimental validation and continued investigation of nitroindazole derivatives as promising therapeutic candidates.

## 1. Introduction

Neglected tropical diseases (NTDs) constitute a diverse group of infectious diseases caused by various agents, including viruses, bacteria, parasites, fungi, and toxins [1,2,3,4]. These diseases impose substantial economic, health, and social burdens, disproportionately affecting impoverished communities, and are distributed across vast geographic regions. Many NTDs involve transmission through vectors with complex biological life cycles, complicating disease management and treatment strategies. For instance, leishmaniasis is an NTD caused by protozoan parasites of the genus *Leishmania*, transmitted to humans by the bite of infected female phlebotomine sandflies [5]. It is endemic in more than 98 countries across four continents, including the Americas, Africa, Asia, and parts of Europe. The World Health Organization estimates that each year approximately 1.3 million new cases occur, of which about 300,000 correspond to the visceral form, the most severe and potentially fatal clinical presentation if untreated [6]. Beyond its medical impact, leishmaniasis imposes a significant socioeconomic burden, particularly in low- and middle-income countries, where the disease is strongly associated with poverty, poor housing conditions, and limited access to healthcare [7].

Current therapeutic strategies for leishmaniasis encompass a variety of approaches, including drug therapy, nanoparticles, vaccines and immunotherapies [8,9,10]. However, these therapeutic options frequently exhibit significant limitations, such as toxicity, insufficient efficacy, and high treatment costs [11]. Commonly employed pharmacological agents include pentamidine, paromomycin, miltefosine, amphotericin B, and pentavalent antimonial derivatives [12,13]. However, prolonged and repeated exposure to these drugs has led to parasite resistance, attributed to long treatment durations and variable efficacy across different *Leishmania* species [13,14]. Consequently, there is an urgent need for the development and exploration of novel therapeutic agents that offer greater safety, improved efficacy, and reduced risk of resistance in the treatment of leishmaniasis.

**Figure 1:**
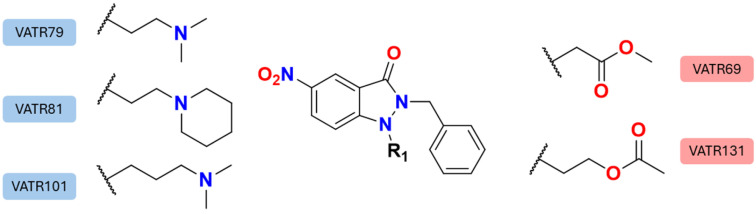
Structure of nitroindazole derivatives as potential antileishmanial agents.

Nitroindazole derivatives have emerged as promising therapeutic candidates for the treatment of protozoan diseases such as Chagas disease, trichomoniasis, and leishmaniasis [15]. Recent in vitro studies have demonstrated that these compounds exhibit significant selectivity and potency against *Leishmania amazonensis*, with eight derivatives displaying high selectivity indices and submicromolar IC50 values (<1 μM), and four compounds showing activity comparable to the reference drug, Amphotericin B (AmB). These findings led Mollineda et al. to propose nitroindazole derivatives as potential therapeutic agents for leishmaniasis [16] (some structures are presented in Figure 1). Additionally, electron microscopy analyses have revealed that these compounds induce pronounced structural damage to the parasite, further supporting their therapeutic potential [17].

Among the previously evaluated nitroindazole derivatives, VATR131 exhibited low toxicity, a mean inhibitory concentration lower than 10 µM and a selectivity index of 875 against *L. amazonensis* [16]. In addition, electronic microscopy studies confirmed VATR131’s leishmanicidal activity, causing both structural and ultrastructural damage to the parasite [17]. These findings support VATR131 as a promising candidate for further investigations as a potential antileishmanial hit.

On the other hand, computational tools have become essential in drug discovery pipelines. Their applications range from studying the dynamic nature of biological systems to virtual screening and the discovery of bioactive compounds [18,19,20]. Other applications of computational tools include modeling the potential mechanisms of action of bioactive compounds lacking experimental validation. To bridge this gap, recent studies have focused on elucidating the mechanisms of action of compounds with promising bioactivity. Computational approaches, including target fishing, molecular docking, and molecular dynamics simulations, have been employed extensively for this purpose.

For instance, the study conducted by Steverding et al. [21] utilized these computational strategies to identify potential molecular targets and mechanisms of action of gallic acid alkyl esters against *Trypanosoma brucei* and *Leishmania major*. These compounds showed moderate to strong trypanocidal effects, especially esters with 3–5 carbon chains, while leishmanicidal activity was low. The most active compounds were selective and likely act by inhibiting *T. brucei* alternative oxidase, as supported by molecular modeling and rapid parasite immobilization in glycerol-based assays. Similarly, Kovacs et al. [22] reported that the antiprotozoal agent nitazoxanide also acts as an agonist of peroxisome proliferator-activated receptors (PPARs). As a result, the drug was shown to lower blood glucose levels and improve insulin sensitivity in rats with type 1 diabetes, as demonstrated through in vivo studies. In a separate study, Cheng et al. [23] predicted and subsequently confirmed through in vitro assays that montelukast, a cysteinyl leukotriene receptor 1 antagonist, also functions as a dipeptidyl peptidase-IV (DPP-IV) inhibitor.

Based on this background, the present study aims to investigate the activity of VATR131 against *L. infantum* and to propose its potential mechanism of action. To this end, in vitro inhibition studies were conducted on both the amastigote and promastigote forms of *L. infantum*. Computational methodologies, including target fishing, molecular docking, and molecular dynamics simulations were also employed to explore the compound’s potential mechanism of action.

## 2. Results

### 2.1. In Vitro Evaluation

The results of the evaluation of VATR131 and AmB against *L. infantum* amastigotes and promastigotes are presented in Table 1. The table reports the Half Maximal Inhibitory Concentration (IC50), standard deviations (SD) and the Selectivity Indices (SI). The selectivity index is calculated from a 50% Cytotoxicity Concentration (CC50).

### 2.2. Overview of Modeling Studies

Computational modeling studies were conducted to identify the most probable molecular targets of compound VATR131 (2-(benzyl-2,3-dihydro-5-nitro-3-oxoindazol-1-yl) ethyl acetate) against *L. infantum*. Initially, potential molecular targets for VATR131 were predicted using a computational target-fishing approach [24,25,26]. Subsequently, rigid receptor molecular docking calculations were performed for the predicted targets, generating multiple binding hypotheses. To further refine these predictions incorporating protein flexibility and higher accuracy, molecular dynamics (MD) simulations were performed on the docked ligand–protein complexes, and binding free energies were estimated from conformational ensembles extracted from the MD trajectories. While molecular docking serves to generate initial binding hypotheses, the MD-derived binding energies were the criterion for ranking and prioritizing the most likely molecular targets of VATR131 in *L. infantum*. This integrated computational approach provided more accurate and reliable predictions compared to molecular docking alone, as the binding free energies derived from MD simulations helped mitigate biases associated with comparing docking scores across different protein targets [27].

### 2.3. Target Fishing and Homology Models

Potential molecular targets of VATR131 in *L. infantum* were predicted following the computational procedure described in the Materials and Methods section. Specifically, a homology-based computational target-fishing approach was employed, based on the similarity principle and using reference databases of experimentally validated ligand–receptor interactions. Given that these reference databases predominantly include human targets, the homology-based approach was specifically adapted to identify relevant *L. infantum* targets for VATR131. The resulting list of 11 predicted molecular targets obtained through this computational strategy is summarized in Table 2. None of the predicted targets had experimentally determined structures. For this reason, homology models were generated for all of them. Relevant information from the homology modeling process is summarized in Table 2.

### 2.4. Molecular Docking

Molecular docking studies were conducted for VATR131 against the predicted proteins listed in Table 2, following the methodology described in the Materials and Methods section. The docking scores obtained from these calculations for each potential target are summarized in Table 3. The table presents the score from the initial docking run (“Score”), the median score from 50 repetitions with different random seeds, and the corresponding standard deviations. The change in the random seed is performed to account for the stochastic nature of the docking algorithm. A summary of the results obtained in the 50 repetitions of the docking process in each protein is given as Supplementary Material in Figure S1. The low variability in docking scores across the 50 replicas (all with SD < 5%) indicates convergence of the docking calculations. Additionally, the closeness of the docking score of the first (reference) docking run to the median score of the docking replicas justifies the selection of these reference solutions for further investigations.

### 2.5. Molecular Dynamics Simulations

Although molecular docking is widely employed to investigate ligand–receptor interactions, it has inherent limitations, primarily due to the simplified representation of molecular interactions required for computational efficiency. To enhance the reliability and accuracy of molecular modeling predictions, post-processing methods such as molecular dynamics (MD) simulations can be utilized. Previous studies have demonstrated that incorporating MD simulations to estimate the binding free energies of ligand–receptor complexes can significantly improve the prioritization of relevant molecular targets for chemical compounds [24,28,29]. Accordingly, the 11 ligand–protein complexes predicted by molecular docking were further analyzed using the MD simulations protocol described in the Materials and Methods section. Subsequently, the binding free energies of VATR131 to its potential targets were calculated from conformational ensembles extracted from the MD simulations using the Molecular Mechanics Poisson–Boltzmann Surface Area (MM-PBSA) method.

MD simulations are primarily employed in this research to produce conformational ensembles that are used to predict the free energies of binding with the MM-PBSA method. To evaluate the stability of the systems along the MD simulations and the diversity of the conformational sampling, parameters such as Root Mean Square Deviation (RMSD) and Root Mean Square Fluctuations (RMSF) were investigated for the 55 MD production trajectories. Detailed information on the molecular dynamics systems, including atom counts, solvent composition, ionic content, and periodic box dimensions, is provided in Table S2 of the Supporting Information. These results are presented as Supplementary Information in Figures S2–S23. The analysis of the RMSD values shows that the ligand remains stable in the binding cavity, with RMSD values below or close to 2 Å relative to the starting docking conformation. The same behavior is also observed for all receptors, except those of the HSP type: HSP60, HSP60-1, HSP60-2 and HSP60-3. In the later cases, stability is also observed but at the expense of larger deviations from the initial receptor conformation.

To clarify whether these RMSD values could largely change the initial binding hypotheses, the RMSF values of the protein backbone were analyzed (Figures S13–S23). This analysis reveals that in all cases the larger system flexibility is concentrated in regions away from the ligand binding site and that this region is among the least flexible portions of the analyzed proteins. Overall, system flexibility shows stability on the ligand binding region and the ligand, while the different profiles of the RMSD values among MD replicas indicate that different conformational regions are explored. This is precisely one of the advantages of conducting multiple MD replicas over relying on a single trajectory for MM-PBSA calculations.

**Figure 2:**
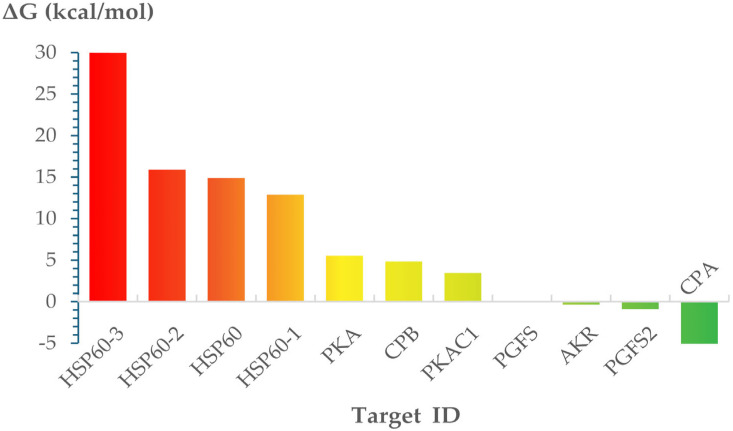
Predicted free energies of binding of 2-(benzyl-2,3-dihydro-5-nitro-3-oxoindazol-1-yl) ethyl acetate (VATR131) to its potential targets in *L. infantum*.

The predicted binding free energies for VATR131 and its potential receptor proteins are presented in Supporting Information Table S1 and summarized in Figure 2. Among the evaluated targets, cysteine peptidase A (CPA) exhibited the lowest (best) binding free energy (−5.04 kcal/mol), indicating the strongest predicted interaction. Based on these findings, the VATR131–CPA complex was selected for more detailed structural analysis.

**Figure 3:**
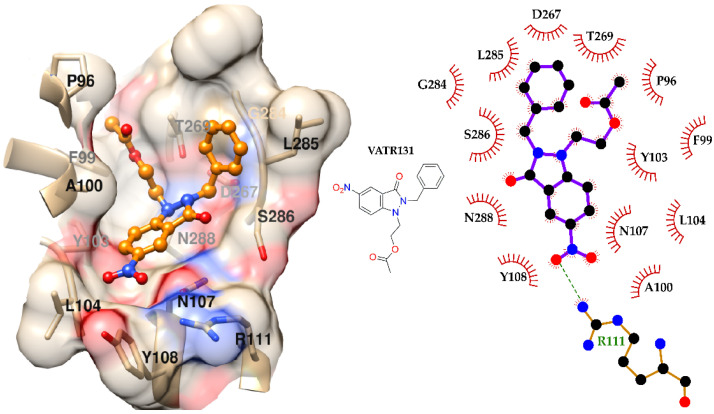
Detailed orientation of the ligand in the binding cavity of CPA, along with a diagram of the predicted ligand–receptor interactions. VATR131 is represented as balls and sticks. Oxygen and nitrogen atoms are red and blue, respectively.

The predicted binding mode of VATR131 within the CPA active site is illustrated in Figure 3. The ligand conformation depicted corresponds to the centroid structure of the most populated cluster, derived from clustering analysis of the 100 snapshots extracted from molecular dynamics (MD) simulations for MM-PBSA calculations. CPA residues interacting with VATR131 in at least 50% of the MD snapshots were identified and labeled in Figure 3.

## 3. Discussion

Compound VATR131, with leishmanicidal activity against *L. amazonensis*, was selected in the present study for evaluation against *L. infantum* and for investigation of its potential mechanism of action. As shown in Table 1, VATR131 demonstrated efficacy against both the promastigote and intracellular amastigote forms of *L. infantum*. The compound exhibited SI greater than 10, and its selectivity against amastigotes surpassed that of the reference drug AmB.

For the in vitro assays, the resazurin transformation method was selected because of its higher reproducibility and reliability compared to manual microscopic counting. Fluorimetric assays minimize operator bias and provide robust quantification of intracellular amastigotes, representing a validated alternative to traditional microscopy [30].

Previous studies using the resazurin transformation method have reported that *L. amazonensis* is more sensitive than *L. infantum* to 3-alkoxy-1-benzyl-5-nitroindazole derivatives. This trend is consistent with the recognized refractoriness of visceral leishmaniasis and the variable efficacy of current treatments across different *Leishmania* species. Accordingly, the variability observed in our study reflects biological differences in drug susceptibility between species, rather than experimental divergence [31]. Likewise, the differences in IC_50_ values observed between *Leishmania* species should not be attributed to methodological inconsistencies, but rather to intrinsic species-specific susceptibility.

According to the results of molecular docking, the targets displaying the most favorable docking scores were proteins HSP60-2, HSP60-3, and PKA, with binding energies of −9.19, −9.19, and −8.64 kcal/mol, respectively. Conversely, the least favorable docking scores were observed for proteins CPB, CPA, and PGFS2, showing values of −6.34, −5.78, and −6.97 kcal/mol, respectively. Despite these differences in docking scores, visual inspection of the ligand–protein complexes revealed potentially favorable interactions in all cases, with VATR131 appropriately occupying the binding sites of each receptor. The observed interactions were predominantly hydrophobic and Van der Waals in nature. Nevertheless, docking scores alone were insufficient to confidently prioritize a subset of the 11 potential targets evaluated, highlighting the need for further analyses.

As a result of the MM-PBSA studies, the CPA complex had the best (lowest) binding free energy. In the complex of VATR131 with CPA, several interactions are present. The nitro group of VATR131 is predicted to form a hydrogen bond with residue R111, mediated by the guanidine moiety of the arginine side chain. In this interaction, the side chain of R111 acts as a hydrogen bond donor, while one oxygen atom of the nitro group serves as the acceptor. This hydrogen bond contributes significantly to the orientation of the ligand in active site and, in combination with hydrophobic and van der Waals interactions, is key in the formation of the ligand–protein complex. Additionally, previous studies have reported that nitro groups can participate in non-conventional hydrogen bonding interactions, involving hydrogen atoms attached to sp^3^ carbons [32]. Consistent with this observation, the hydrogen atoms of residue L104 might interact with the second oxygen atom of the nitro group, further stabilizing the complex, an effect that is likely facilitated by the electrostatic potentials associated with this substituent [33,34] (see Figure 3).

Moreover, a π-alkyl interaction was observed between residue A100 and the benzene ring of the 5-nitroindazole moiety. Specifically, this interaction occurs between the methyl group of A100 and the π-electron cloud of the benzene ring [35]. Additionally, hydrophobic interactions were identified between S286 and the methylene hydrogens linking the 5-nitroindazole core to its benzyl substituent. These hydrophobic contacts likely contribute to the exclusion of water molecules from the binding site, which is situated within a predominantly hydrophobic region. Furthermore, residue D267 might interact through a hydrogen bond with the methylene hydrogens adjacent to the 5-nitroindazole core. This interaction arises from the proximity of the carboxyl group of D267 to the methylene hydrogens, a scenario similar to that described for residue L104. The formation of this hydrogen bond is likely facilitated by the nearby electro-negative nitrogen atom within the 5-nitroindazole ring, which increases the partial positive character of the methylene hydrogens, making them energetically favorable for hydrogen bond formation.

The isobutyl group of residue L285 forms a π-alkyl interaction with the π-electron cloud of the benzene ring in VATR131, a phenomenon similar to the interaction observed with residue A100 and consistent with findings reported by Vaidyanatham and collaborators [36]. Additionally, several van der Waals interactions were identified between VATR131 and residues of target. Apolar–apolar interaction was found for P96, F283 and F99, polar-polar interaction for Y108, N107, T269 and Y103, and polar–apolar interaction for N288 between residues and VATR131, respectively. Collectively, these interactions, along with hydrogen bonding and π-alkyl interactions, likely enhance the binding affinity between the protein and the ligand, contributing to the stabilization of the complex. Interestingly, the ester group of VATR131, located at a branching point of the molecule, does not appear to engage in significant interactions with the surrounding protein residues. This suggests that the ester group is relatively free to move within the binding environment, potentially contributing to the flexibility of the ligand within the binding cavity.

In the study conducted by Mollineda et al. [16], several compounds from the VATR series were evaluated. In this series, all compounds share a common scaffold featuring a nitro group and a benzyl substituent, while the side chain constitutes the main source of structural variation. Compounds with lower desirability indices (VATR79, 81, and 101) contain tertiary amines, which confer increased hydrophobic character compared to the ester-containing VATR131 and VATR69. These observations suggest that the binding mode across the VATR series, as well as the key interactions with residues R111, L104, A100, S286, and D267, are likely conserved due to the shared scaffold. Moreover, in cases where the side chain exhibits greater hydrophobicity, the compound tends to display lower predicted binding energy toward the target CPA, which may correlate with reduced inhibitory activity against the parasite.

Numerous studies have demonstrated that cysteine peptidases are critically involved in the survival, replication, and pathogenesis of parasitic protozoa [37,38,39,40]. Owing to their essential biological functions, these enzymes have been identified as attractive targets for drug development and vaccine design aimed at disease control [41]. Their proteolytic activity enables the parasite to degrade host proteins, facilitating nutrient acquisition necessary for its growth and persistence [42]. Additionally, by degrading immune-related proteins in the host environment, cysteine peptidases contribute to immune evasion [43,44,45]. Efforts to identify inhibitors of these enzymes have shown promise as inhibition of cysteine peptidase activity can either lead to parasite death [46] or significantly weaken the parasite by disrupting its interaction with the host and attenuating its virulence [38]. This, in turn, increases its susceptibility to immune clearance and contributes to the elimination of the infection [47]. Taken together, the computational models suggest that the molecular target of the nitroindazole derivatives, particularly VATR131, is the CPA enzyme, which plays a critical role in the survival and drug resistance of the *Leishmania* parasite. Previous studies have shown that CPA acts as a virulence factor without affecting promastigote growth. Nonetheless, selective cysteine protease inhibitors have been reported to kill *Leishmania* in vitro at non-toxic concentrations for host cells [46], reinforcing the therapeutic potential of targeting cysteine proteases, including CPA. Nevertheless, further experimental studies are required to evaluate the proposed hypotheses.

In a previous study [17], the mechanism of action of nitroindazole derivatives was not directly demonstrated but rather hypothesized from ultrastructural alterations observed by electron microscopy. Although the generation of reactive oxygen species (ROS) may contribute to parasite damage, the precise mechanism of action of these compounds remained undefined. The present study addresses this gap by proposing CPA as a plausible molecular target of VATR131. Studies in the related parasite *Trypanosoma cruzi* demonstrated that cysteine protease inhibitors can block normal autoproteolytic processing of cruzain in the Golgi complex, interrupting the life cycle in vitro and in vivo, while sparing host cell organelles [48,49]. Therefore, while ROS generation cannot be completely excluded as an additional mechanism of action, our results strongly support CPA inhibition as a central and specific pathway underlying VATR131 activity.

Optimally, the results obtained should be compared with the application of the same methodologies to known binders of the studied proteins. However, no compounds have yet been reported to bind any of the modeled proteins. Thus, comparisons between our results and benchmarking experiments are not possible, which represents a limitation of our work together with the lack of experimental CPA inhibition assays. In this sense, we considered that using MD-derived binding energies for prioritizing the likely targets of VATR131 in *L. infantum* provides a valid starting point for future experimental validations.

## 4. Materials and Methods

### 4.1. Experimental Methods

#### 4.1.1. Chemistry

For the in vitro assays, the compound VATR131 was dissolved in dimethyl sulfoxide (DMSO, Sigma-Aldrich, St. Louis, MO, USA) at initial concentrations of 1 and 10 mg/mL. AmB sodium deoxycholate was used as a positive control in all experiments (Julio Trigo López Pharmaceutical Laboratory Company, Havana, Cuba) and was dissolved in sterile distilled water at a concentration of 2 mg/mL and stored at 4 °C until its time of use in each experiment.

#### 4.1.2. Parasites

The experiments employed the *L. infantum* strain MHOM/FR/78/LEM75, kindly provided by the Department of Parasitology, Faculty of Pharmacy, Complutense University of Madrid (Spain). Promastigotes were isolated from skin lesions of infected BALB/c mice and cultured at 26 °C in Schneider’s medium (Sigma-Aldrich, St. Louis, MO, USA) supplemented with 10% heat-inactivated fetal bovine serum (FBS; 56 °C for 30 min), penicillin (200 IU/mL), and streptomycin (200 µg/mL). To ensure exponential growth, the cultures were subcultured every 3–4 days. Parasites were used only when passage number was below ten.

#### 4.1.3. Susceptibility Assay in Promastigotes

Exponentially growing promastigotes were seeded in 96-well plates at a density of 1 × 10^6^ cells/mL in a final volume of 200 µL per well. VATR131 was tested at eight concentrations prepared through serial 1:2.5 dilutions, covering a range from 130 to 0.2 µM. Cultures were incubated at 26 °C for 72 h before viability assessment.

#### 4.1.4. Peritoneal Macrophage Extraction

Resident macrophages were collected from BALB/c mice via peritoneal lavage following euthanasia by cervical dislocation. The peritoneal cavity was washed with cold (4 °C) RPMI 1640 medium (Roswell Park Memorial Institute, Buffalo, NY, USA) supplemented with 10% FBS and antibiotics (penicillin 200 IU/mL and streptomycin 200 µg/mL). Cell concentration was determined by manual counting in a Neubauer chamber under 400× magnification using a Motic optical microscope (Xiamen, China).

#### 4.1.5. Cytotoxicity in Mouse Peritoneal Macrophages

Macrophages were adjusted to a concentration of 3 × 10^5^ cells/mL, and 200 µL of the suspension were seeded per well in 96-well plates. After 2 h of incubation at 33 °C and 5% CO_2_ to allow adhesion, non-adherent cells were removed by washing with phosphate-buffered saline (PBS). Cells were then treated with VATR131 at a maximum concentration of 650 µM, followed by seven serial dilutions (1:2.5 ratio). Each concentration was tested in quadruplicate. After 48 h, 20 µL of a 3 mM resazurin solution were added to each well. Following 4 h of incubation, fluorescence was measured using a plate reader (SUMA, Ultra Microanalytical System, Cuba). Cytotoxicity was assessed by determining the 50% cytotoxic concentration (CC_50_) from the non-linear regression of fluorescence versus concentration curves. Each assay was independently replicated at least three times.

#### 4.1.6. Intracellular Amastigote Assay

Macrophages were seeded at 1 × 10^5^ cells/mL in 96-well plates, with a final volume of 200 µL per well, and incubated for 2 h at 33 °C under 5% CO_2_. After cell adhesion, stationary-phase promastigotes were added at a ratio of 10:1 (parasites: macrophages), followed by 4 h of co-incubation. The wells were then treated with VATR131 at concentrations ranging from 15 to 0.02 µM using a 1:2.5 dilution scheme. After 48 h of treatment, RPMI medium was replaced with Schneider’s medium, and the plates were incubated at 26 °C for an additional 72 h to allow surviving amastigotes to differentiate and replicate as promastigotes [50].

Positive (AmB) and negative (0.1% DMSO) controls were included in all assays. Each concentration was tested in quadruplicate. Cell viability was assessed using the resazurin transformation method [51]. Specifically, 20 µL of 3 mM resazurin solution prepared in sterile PBS were added to each well, and fluorescence was read after 8 h at excitation/emission wavelengths of 540/590 nm using a SUMA^®^ microplate reader (Habana, Cuba).

The mean fluorescence intensity for each condition was calculated and used to fit a sigmoidal Emax model [52]. From this, the half-maximal inhibitory concentration (IC_50_) values were derived for promastigotes and intracellular amastigotes. The selectivity index (SI) was calculated as the ratio of CC_50_ to IC_50_.

### 4.2. Computational Methods

This study applied a multistep computational protocol to investigate the potential molecular mechanism of action of the nitroindazole derivative VATR131 against *L. infantum*. The workflow included (i) target fishing using a homology-based approach to identify parasite proteins potentially interacting with VATR131; (ii) molecular docking to predict binding modes and affinities of the ligand to each target; and (iii) molecular dynamics simulations combined with MM-PBSA binding free energy calculations to assess the structural stability and interaction energetics of the ligand–receptor complexes. Otherwise noted, default parameters were used in the calculations described below.

#### 4.2.1. Target Selection

Potential molecular targets for VATR131 were identified using a previously described homology-based target fishing approach [53,54]. Initially, candidate targets were predicted using the Similarity Ensemble Approach (SEA) web server [55]. The resulting predicted targets were subsequently used as queries in a BLAST 2.16.0 search [56] against the *L. infantum* (taxid: 5671) proteins available in the Reference Sequence Protein (RefSeq Protein) database. This search was performed using the NCBI BLAST server (https://blast.ncbi.nlm.nih.gov/, accessed on 2 June 2025). Proteins from *L. infantum* were considered potential targets if the BLAST alignments demonstrated a minimum sequence identity of 35% and a sequence coverage of at least 70%.

#### 4.2.2. Molecular Docking

The initial three-dimensional structure of VATR131 was generated using the OpenEye Omega 6.0.0 software [57], and AM1-BCC partial atomic charges were assigned using MolCharge 2.2.5 [58]. The formal charge of the compound was set to 0. Since experimentally determined three-dimensional structures were unavailable for the predicted protein targets, homology models were constructed for each protein using the SWISS-MODEL server [59]. Functionally relevant cofactors were incorporated into these homology models based on their respective template structures to ensure accurate representation of the binding sites.

The binding sites of the receptor proteins were defined based on the positions of ligands present in the template structures used for homology modeling. Binding site residues were defined as those located within 6 Å of the reference ligand.

Molecular docking studies between VATR131 and the target proteins were per-formed using AutoDock Tools 1.5.7 and AutoDock Vina 1.2.3 [60]. Ligand preparation involved loading VATR131 into AutoDock Tools, identifying the ligand root, setting the number of torsions to seven, and saving the ligand structure in pdbqt format. Target protein structures were prepared by adding polar hydrogen atoms and converting them into pdbqt format. Docking grid boxes were defined by specifying appropriate three-dimensional coordinates (x, y, z) around the identified binding sites. Docking simulations were performed following standard protocols, considering the ligand as flexible [60]. A total of 50 docking replicas were carried out by varying the random seed, which allowed the exploration of multiple docking solutions. Docking results were analyzed using Biovia Discovery Studio 24.1.0 [61] and visualized with PyMOL 2.6.2 [62]. The best-docked conformations for each ligand-receptor complex were selected based on their calculated binding energies (kcal/mol).

#### 4.2.3. Molecular Dynamics Simulations

Molecular dynamics (MD) simulations and Molecular Mechanics Poisson-Boltzmann Surface Area (MM-PBSA) calculations were performed using Amber 20 software [63]. The computational procedures followed the protocol previously described by our research group [53,64]. All predicted ligand–receptor complexes underwent a standardized MD simulation workflow, including energy minimization, heating, equilibration, production runs, and subsequent calculation of binding free energies. The Amber ff14SB force field was employed to parameterize amino acid residues, while the general Amber force field (GAFF) was utilized for non-amino acid components. MD simulations were conducted in explicit solvent conditions, with each ligand–protein complex enclosed within truncated octahedral boxes solvated by TIP3P water molecules. To neutralize net charges and mimic physiological ionic strength, Na^+^ or Cl^-^ ions were added to achieve a final ionic concentration of 0.15 M. For histidine, all residues were considered neutral and automatically handled by tleap during MD simulation setup, assuming the standard uncharged imidazole state at physiological pH.

The prepared systems underwent energy minimization in two sequential steps. Initially, energy minimization was performed using 500 steps of the steepest descent algorithm followed by 500 cycles of conjugate gradient minimization, applying position-al restraints (500 kcal/mol·Å^2^) to all solute atoms. Subsequently, positional restraints were removed, and an additional minimization was conducted using 500 steps of steepest descent followed by 1000 cycles of conjugate gradient minimization without constraints. Long-range electrostatic interactions were calculated using the Particle Mesh Ewald (PME) method implemented in Amber, employing a cutoff distance of 12 Å during all minimization steps.

Following minimization, the systems were gradually heated from 0 K to 300 K over a period of 20 ps under constant volume conditions. During this heating phase, positional restraints were applied to the solute atoms with a force constant of 10 kcal/mol·Å^2^. Temperature regulation was achieved using a Langevin thermostat with a collision frequency of 1.0 ps^−1^, and the PME cutoff was set to 10 Å. Throughout all subsequent molecular dynamics (MD) simulation steps, bonds involving hydrogen atoms were constrained using the SHAKE algorithm, allowing for a larger integration time step and improved computational efficiency.

After the heating step, the systems were equilibrated for 100 ps under constant pressure (1 bar) and temperature (300 K). During equilibration, temperature was maintained using a Langevin thermostat, and pressure was regulated through isotropic position scaling with a relaxation time of 2 ps. The Particle Mesh Ewald (PME) cutoff was set to 10 Å throughout this stage. Subsequently, the equilibrated systems served as starting points for five independent production runs, each lasting 4 ns, employing identical simulation parameters as those used during equilibration. Atomic velocities were randomly initialized at the beginning of each production run.

The binding free energies of VATR131 to its potential receptor proteins were estimated using MM-PBSA calculations, performed with the MM-PBSA.py script provided in Amber20 [65]. For these calculations, 100 snapshots (one snapshot every 40 ps) were extracted from the five production runs, considering the 1 ns–4 ns time interval. The ionic strength for MM-PBSA calculations was set to 150 mM.

Structural representations of the ligand–protein complex were generated using UCSF Chimera 1.19 [66], while ligand–receptor interaction networks were analyzed using Cytoscape 3.9.1 [67], and detailed interaction diagrams were produced with LigPlot+ 2.3 [68]. For RMSD and RMSF analyses, MD trajectories were processed with the cpptraj tool of Amber [69].

## 5. Conclusions

This study assessed the potential of VATR131 as a therapeutic candidate for leishmaniasis. Computational modeling approaches, including target fishing and molecular docking, led to the identification of several putative targets in *Leishmania infantum*. Among them, cysteine peptidase A (CPA) emerged as the most promising based on binding free energy estimations derived from molecular dynamics (MD) simulations, with a calculated ΔG of −5.04 kcal/mol. These results reinforce the docking-based predictions and provide a more reliable evaluation of binding affinity.

Detailed interaction analyses revealed that hydrophobic contacts, π-alkyl stacking, and hydrogen bonding collectively contribute to the stability of the VATR131–CPA complex. These interactions suggest a plausible mechanism of action whereby VATR131 interferes with the proteolytic function of CPA, potentially impairing the parasite’s capacity to evade host immune defenses and to acquire essential nutrients within macrophage cells.

While computational models are inherently limited by their simplifications and predictive uncertainty, the findings presented here offer valuable insights that support the continued exploration of nitroindazole scaffolds. In particular, VATR131 stands out as a strong candidate for further development, complementing previous studies in the field. Nevertheless, experimental validation remains essential to confirm the predicted interactions and assess the translational potential of this compound.

Ultimately, the growing resistance to current treatments and the toxicity associated with existing drugs highlight the urgent need to develop safer and more effective therapies for leishmaniasis, an objective for which VATR131 and related compounds may represent promising leads. Altogether, these findings support the continued investigation of nitroindazole derivatives as viable candidates for leishmaniasis chemotherapy.

## Figures and Tables

**Table 1:** Activity of VATR131 and AmB against *L. infantum* amastigotes and promastigotes.

	Promastigotes		Amastigotes		CC_50_ ± SD (µM)
Compound	IC_50_ ± SD (µM)	SI	IC_50_ ± SD (µM)	SI	
VATR131	11.35 ± 1.04	35	10.31 ± 1.4	39	402.6 ± 13.4
AmB	0.28± 0.01	21	0.6 ± 0.21	10	

**Table 2:** Results obtained from the target fishing and homology modeling approaches. The UniProtKB code, PDB templates used for homology models, QMEANDisCo and GMQE quality metrics, query sequence coverage by the template, and sequence identity between the query sequence and the homology model template are presented for all targets.

UniProtKB Entry Name	Target ID ^(a)^	Description	PDB Template	Coverage ^(b)^	QMEANDisCo	QMEAN Z-Scores	GMQE ^(c)^	Sequence Identity (%)
A0A6L0WJ22_LEIIN	CPB	Cathepsin L protease	7AVM	0.70	0.80 ± 0.05	−1.33	0.58	52.6
A4HXY2_LEIIN	PKAC1	cAMP-dependent protein kinase	6F14	0.90	0.83 ± 0.05	−1.00	0.79	50.79
A4HYH2_LEIIN	CPA	Cysteine peptidase A	7AVM	0.86	0.77 ± 0.05	−2.06	0.73	48.69
A4I342_LEIIN	AKR	Aldose reductase	2PDF	0.84	0.71 ± 0.05	−1.67	0.66	38.59
A4I5W0_LEIIN	HSP60	Chaperonin HSP60	4V4O	0.97	0.73 ± 0.05	−1.30	0.75	39.46
A4I6Z4_LEIIN	PGFS	Prostaglandin f2-alpha synthase	4F40	1.00	0.93 ± 0.05	0.03	0.97	95.42
A4I7L0_LEIIN	PGFS2	Prostaglandin F synthase	4GIE	0.96	0.85 ± 0.05	0.17	0.88	58.52
A4I800_LEIIN	HSP60-1	Chaperonin HSP60	4V4O	0.89	0.76 ± 0.05	−0.96	0.73	50.94
A4IBT4_LEIIN	PKA	Protein kinase A	3OVV	0.83	0.80 ± 0.05	−0.86	0.72	52.52
A4IDH4_LEIIN	HSP60-2	Chaperonin HSP60	4V4O	0.94	0.76 ± 0.05	−0.58	0.77	48.59
A4IDH5_LEIIN	HSP60-3	Chaperonin HSP60	5OPX	0.97	0.76 ± 0.05	−0.49	0.78	50.73

^(a)^ ID of each target along the manuscript. ^(b)^ Coverage of the query sequence by the template. ^(c)^ Global Model Quality Estimate.

**Table 3:** Results of docking VATR131 to its potential targets in *L. infantum*.

Target ID	Score (kcal/mol)	Median Score (kcal/mol)	Standard Deviation (kcal/mol)
CPB	−6.34	−6.19	0.28
PKAC1	−8.17	−8.19	0.19
CPA	−5.78	−5.96	0.14
AKR	−7.17	−7.15	0.15
HSP60	−7.76	−7.56	0.18
PGFS	−7.30	−7.37	0.13
PGFS2	−6.97	−7.08	0.12
HSP60-1	−8.60	−8.76	0.30
PKA	−8.64	−8.65	0.28
HSP60-2	−9.19	−9.37	0.36
HSP60-3	−9.19	−9.22	0.34

## Data Availability

The original data presented in the study are included in the article; further inquiries can be directed to the corresponding author.

## Supplementary Materials

The following supporting information can be downloaded at: https://www.mdpi.com/article/10.3390/ph18101489/s1, Figure S1: Boxplot showing the distribution of docking scores obtained from 50 replicas; Figures S2–S12: RMSD plots for the MD production runs. Separate plots are presented for the protein backbone (top) and VATR131 (bottom). Each MD replica is labeled as R1 to R5; Figures S13–S23 Flexibility of protein backbone in the five MD replicas (R1 to R5) for each complex of VATR131 with its potential targets; Table S1: Predicted free energies of binding for VATR131 to its potential targets. Table S2. Composition and characteristics of the molecular dynamics systems simulated in this study.
