# Supplementary material for: Computational Investigation of the Potential Antileishmanial Mechanism of the Nitroindazole Derivative VATR131
Source: Pharmaceuticals (Basel). 2025 Oct 3;18(10):1489. doi: 10.3390/ph18101489 (PMC12567055; doi:10.3390/ph18101489)

Supporting Information for:

Computational investigation of the potential antileishmanial mechanism of the nitroindazole derivative VATR131

Table S1. Predicted free energies of binding for compound VATR131 to its predicted targets are provided, along with the components of these binding energies. All values are expressed in kcal/mol.

Target	MM-PBSA Components							ΔG TOTAL[h]
	VDWaals[a]	EEL[b]	EPB[c]	ENPOLAR[d]	EDISPER[e]	ΔG Gas[f]	ΔG Solv[g]	
CPB	-30.3647	-0.6533	20.9365	-20.3247	35.2399	-31.0180	35.8516	4.8337
PKAC1	-51.0579	-8.0069	38.6979	-34.2784	58.0060	-59.0647	62.5272	3.4625
CPA	-39.8772	-15.5283	33.6979	-26.8214	43.4807	-55.4055	50.3571	-5.0484
AKR	-21.8324	-9.7767	18.7778	-16.0417	28.5230	-31.6091	31.2592	-0.3499
HSP60	-42.0561	-20.3129	53.9221	-28.7560	52.0987	-62.3690	77.2647	14.8957
PGFS	-35.4469	-12.0421	31.1285	-25.7255	42.1824	-47.4890	47.5855	0.0965
PGFS2	-24.4092	-14.5282	24.8368	-17.7412	30.9484	-38.9375	38.0441	-0.8934
HSP60-1	-48.1206	-31.0296	66.2084	-33.6157	59.4301	-79.1502	92.0228	12.8725
PKA	-48.4215	-21.7422	51.5835	-32.5656	56.6941	-70.1637	75.7120	5.5484
HSP60-2	-48.2257	-33.7412	71.7837	-33.1892	59.2737	-81.9669	97.8682	15.9013
HSP60-3	-49.7594	-50.8329	103.8496	-33.1604	61.7095	-100.5923	132.3987	31.8064

(a) van der Waals energy, (b) electrostatic energy, (c) polar solvation energy, (d) non-polar solvation energy, (e) dispersion solvation energy, (f) total gas phase free energy, (g) total solvation free energy, (h) total free energy.

Table S2. Table S2. Composition and characteristics of the molecular dynamics systems simulated in this study. All systems (VATR131 with predicted targets) were solvated in a truncated octahedral periodic box under physiological conditions. The number of atoms of ligand was constant in all systems with 43 atoms.

Predicted	Atoms				Ions		Box dimensions (Å)		
Target	Total	Protein	Water	Cofactor	Na^+	Cl^-	X	Y	Z
CPB	44907	4666	13381	-	19	35	76.46	76.46	76.46
PKAC1	43687	4900	12897	-	23	29	75.81	75.81	75.81
CPA	44715	4650	13323	-	28	26	76.40	76.40	76.40
AKR	40698	4324	12069	73	28	22	73.96	73.96	73.96
HSP60	72881	7878	21624	-	54	34	89.85	89.85	89.85
PGFS	41028	4418	36444	73	28	22	74.09	74.09	74.09
PGFS2	40562	4324	12024	73	28	22	73.90	73.90	73.90
HSP60-1	121048	8053	37600	-	80	72	106.16	106.16	106.16
PKA	49708	5181	14808	-	27	33	79.07	79.07	79.07
HSP60-2	119008	8083	36911	-	79	71	105.58	105.58	105.58
HSP60-3	119925	7999	37244	-	79	72	105.8	105.8	105.8

Figure S1. Boxplot showing the distribution of docking scores obtained from 50 replicas of the docking calculations for all systems.

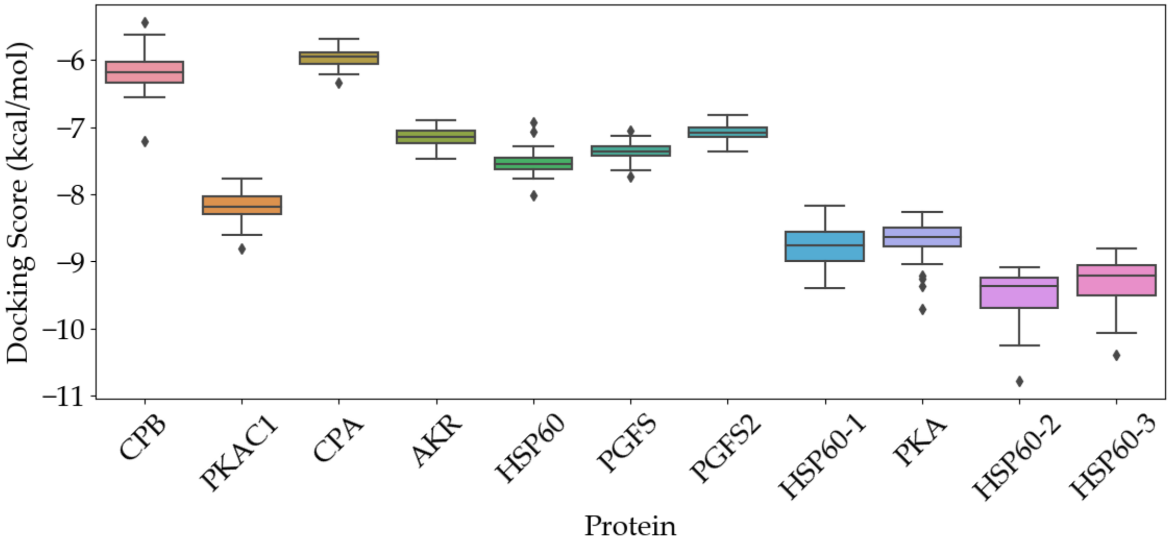

Figure S2. RMSD plots for the complex with CPB. Separate plots are presented for the protein backbone (top) and VATR131 (bottom). Each MD replica is labelled as R1 to R5.

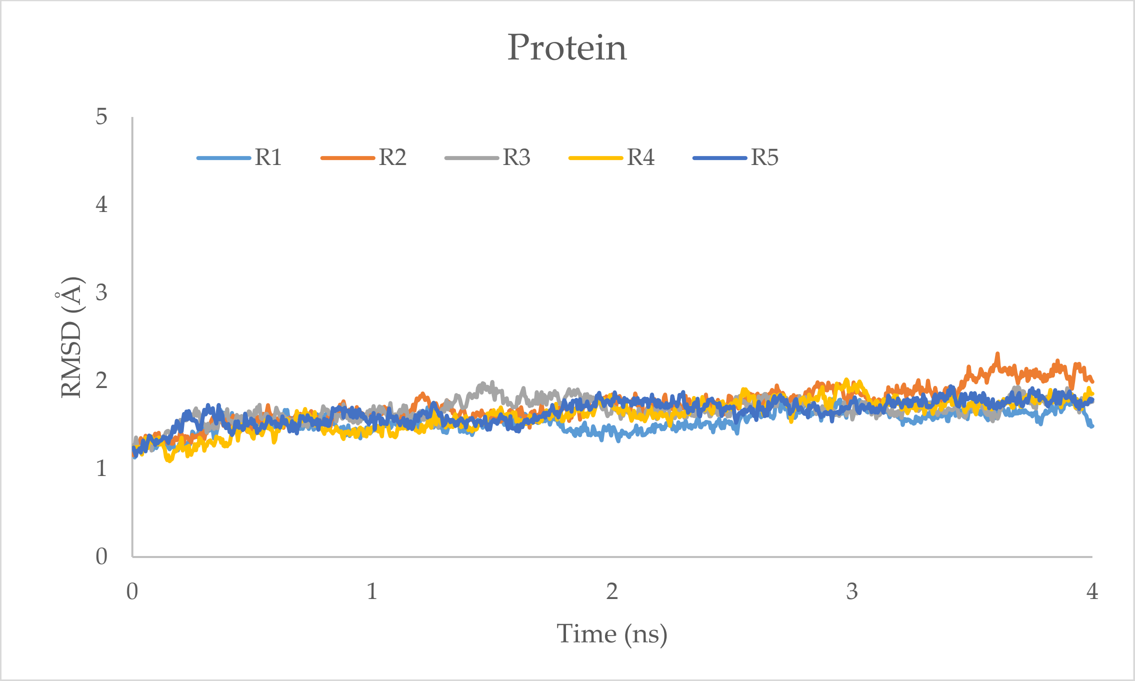

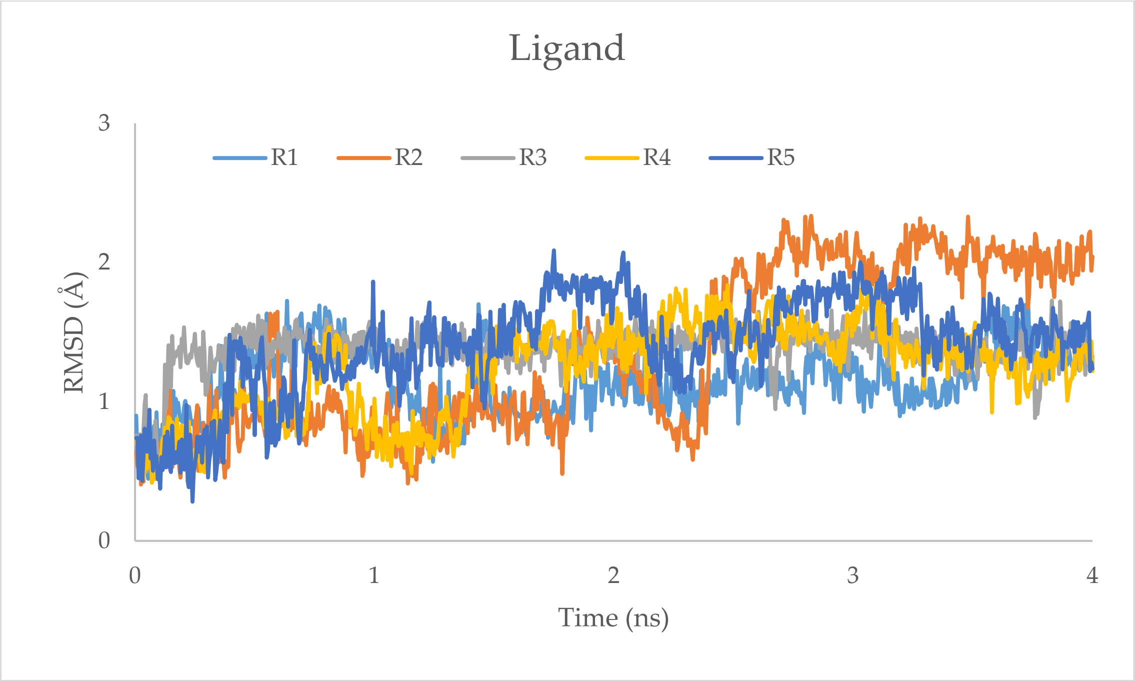

Figure S3. RMSD plots for the complex with PKAC1. Separate plots are presented for the protein backbone (top) and VATR131 (bottom). Each MD replica is labelled as R1 to R5.

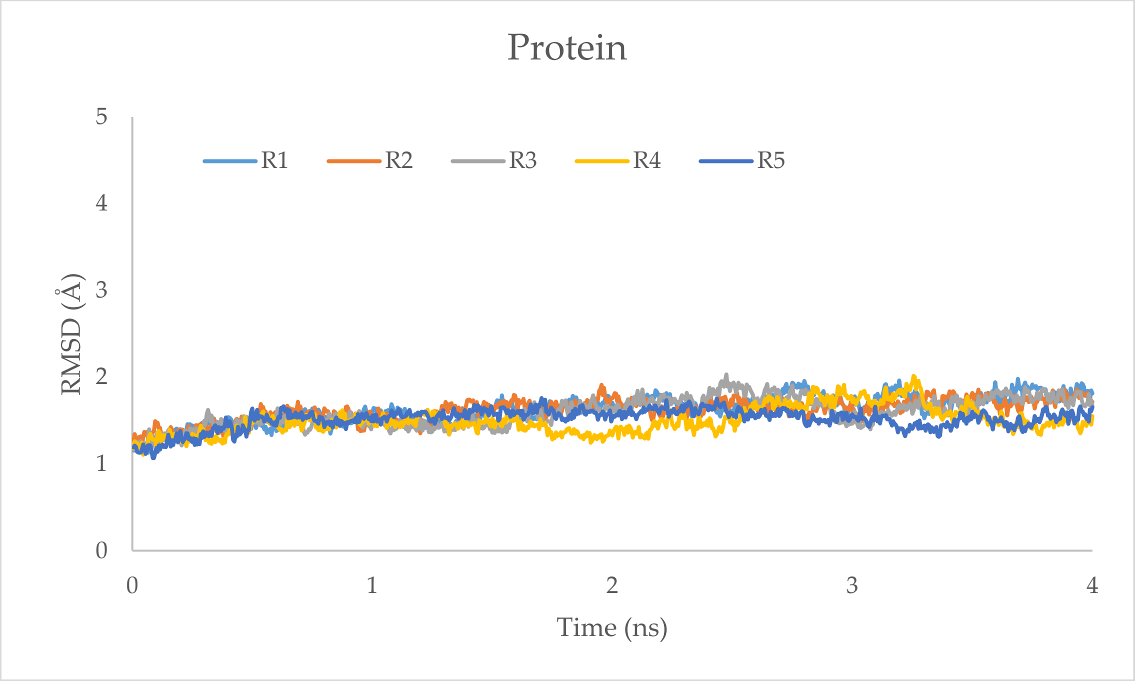

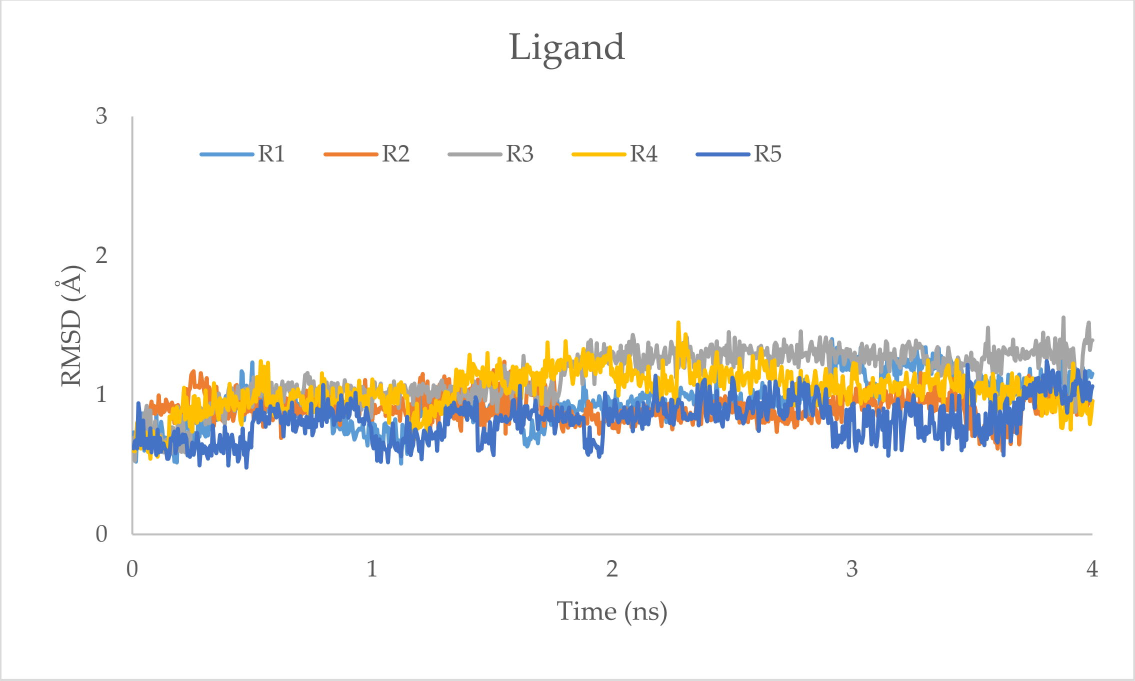

Figure S4. RMSD plots for the complex with CPA. Separate plots are presented for the protein backbone (top) and VATR131 (bottom). Each MD replica is labelled as R1 to R5.

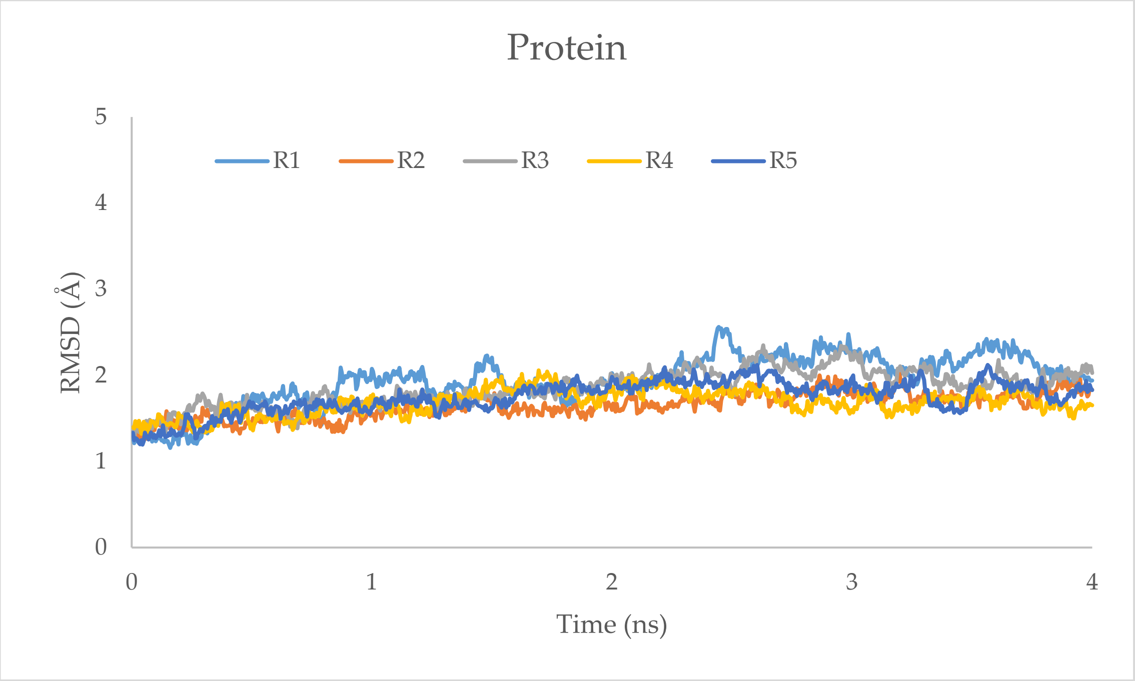

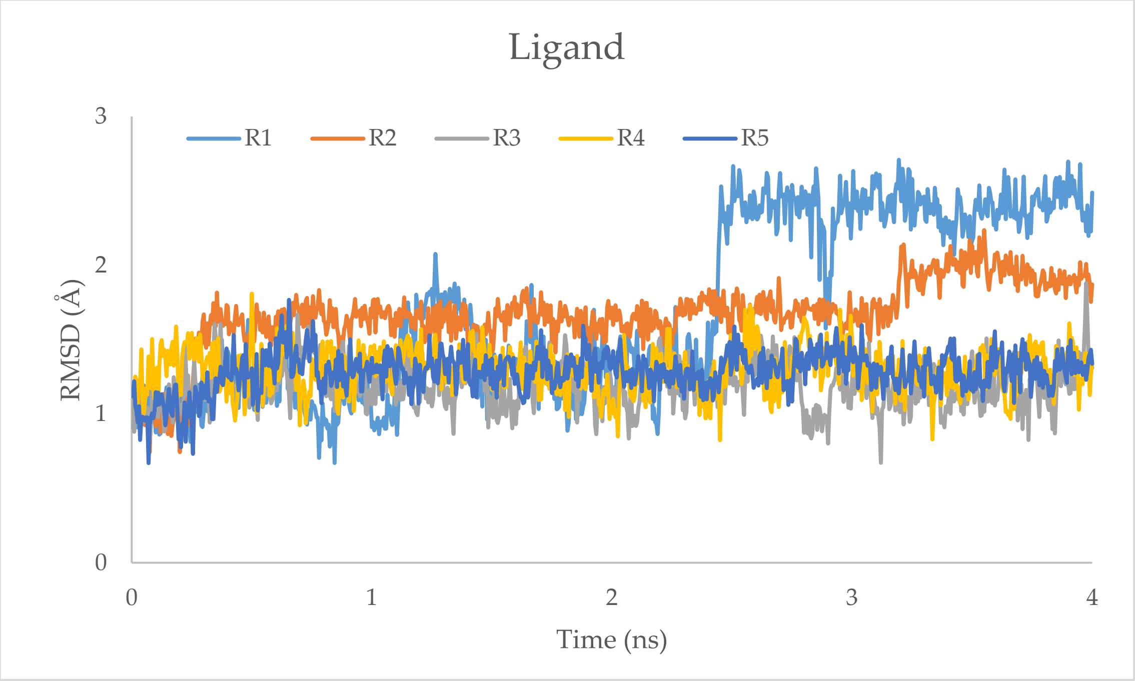

Figure S5. RMSD plots for the complex with HSP60. Separate plots are presented for the protein backbone (top) and VATR131 (bottom). Each MD replica is labelled as R1 to R5.

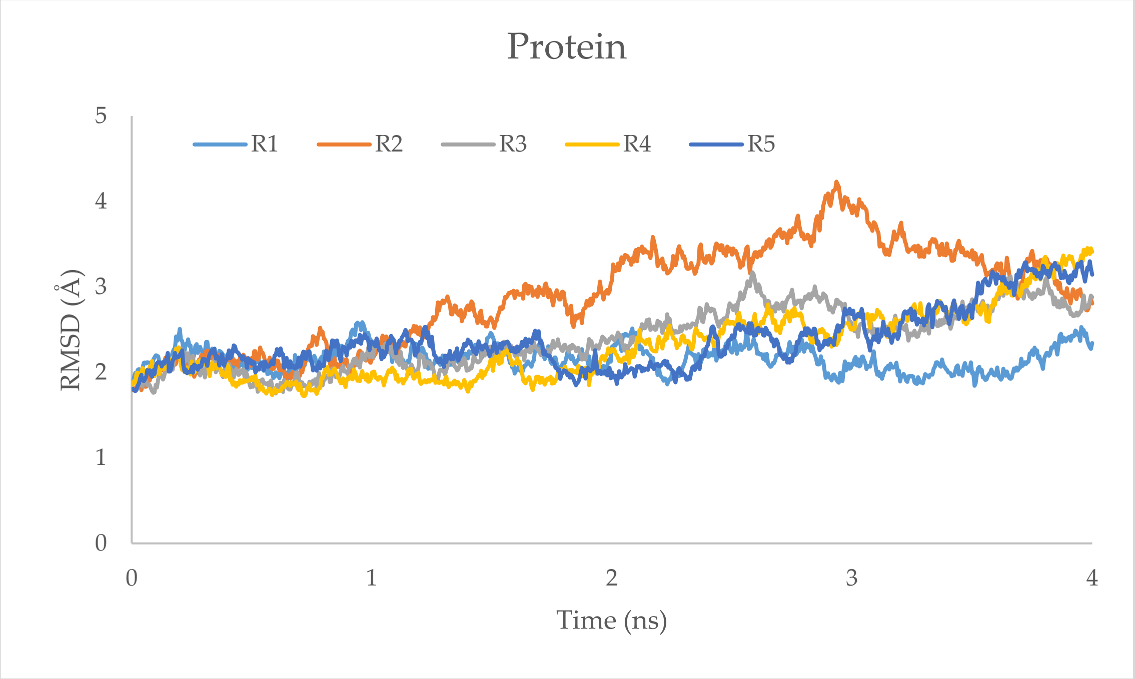

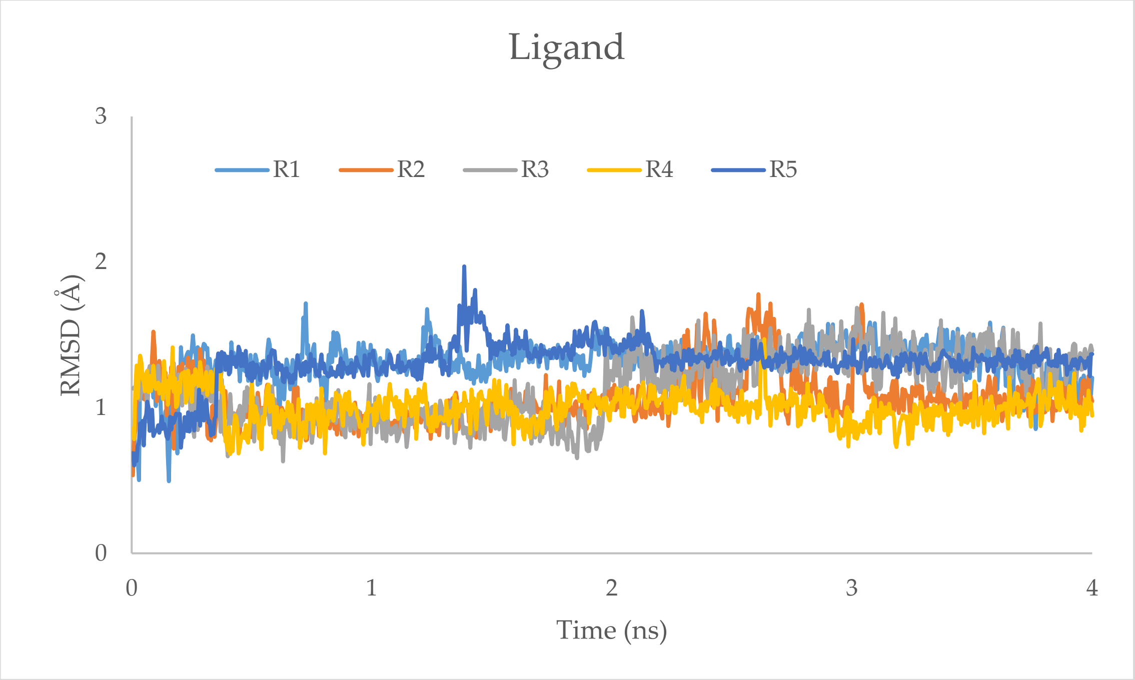

Figure S6. RMSD plots for the complex with PGFS. Separate plots are presented for the protein backbone (top) and VATR131 (bottom). Each MD replica is labelled as R1 to R5.

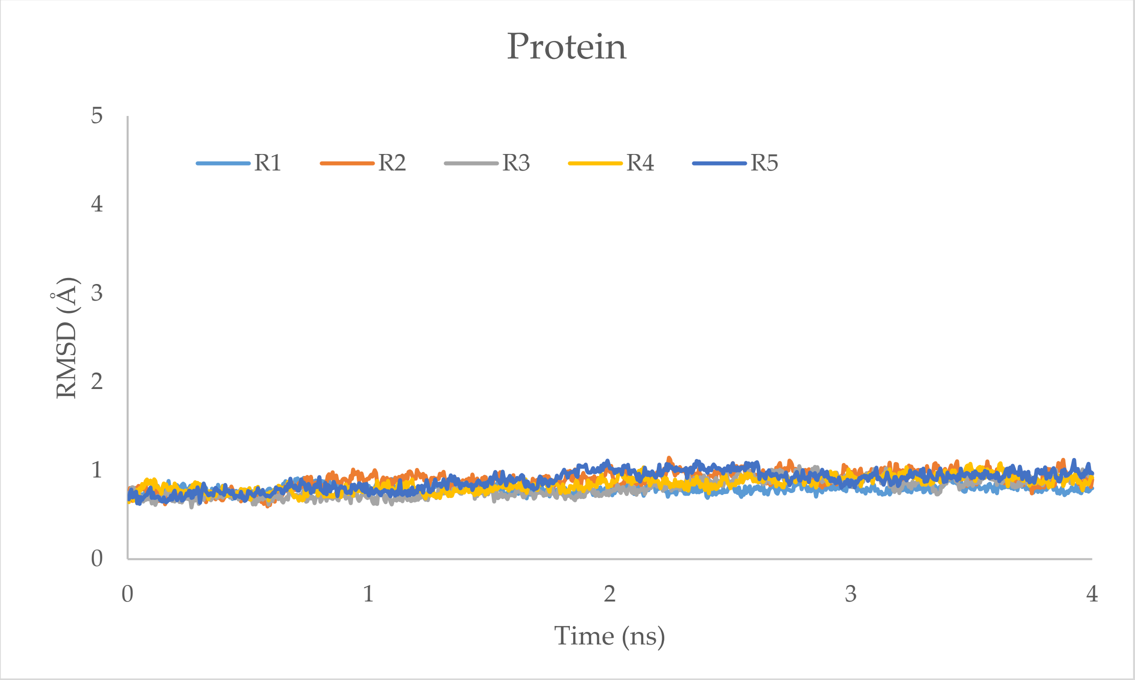

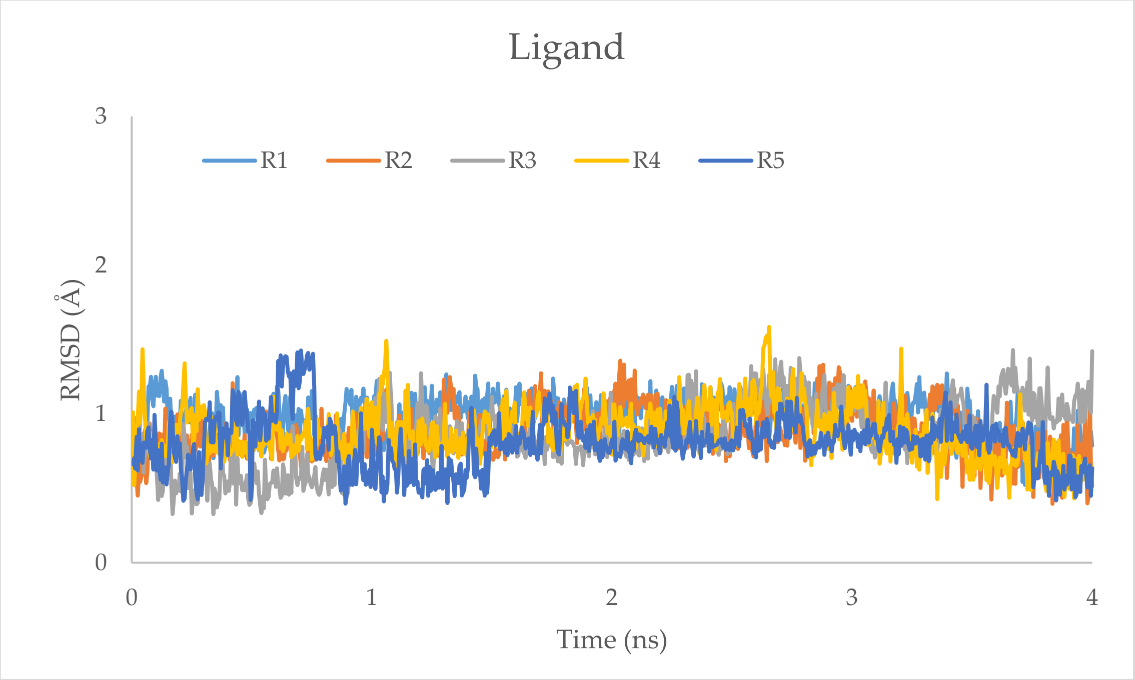

Figure S7. RMSD plots for the complex with PGFS2. Separate plots are presented for the protein backbone (top) and VATR131 (bottom). Each MD replica is labelled as R1 to R5.

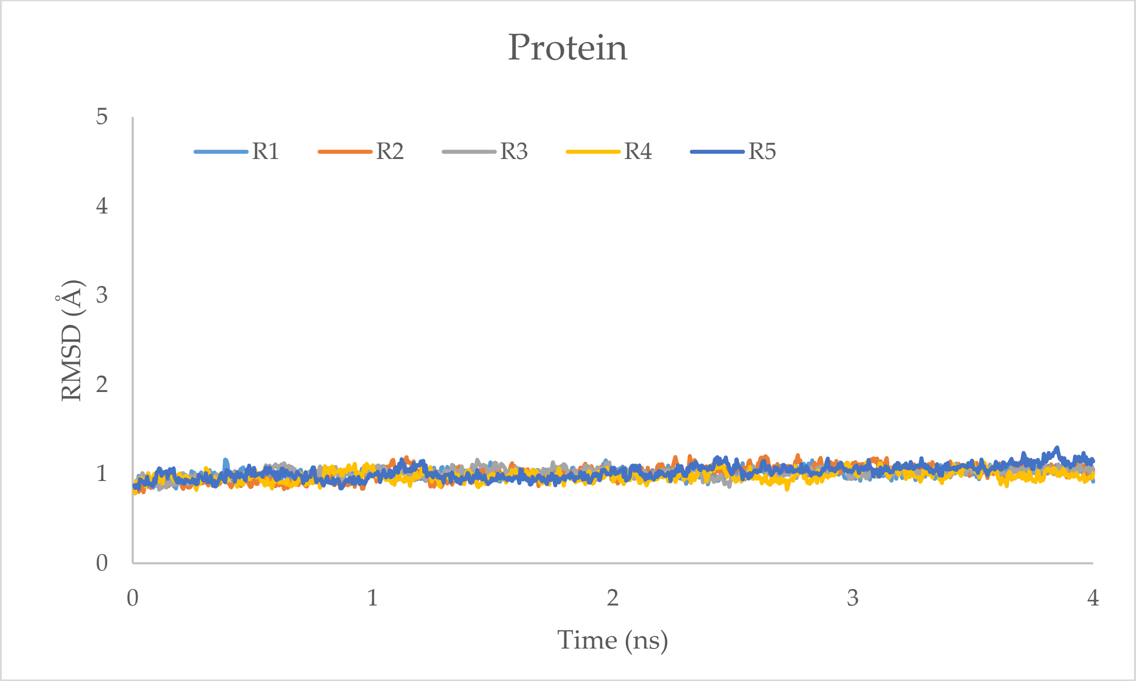

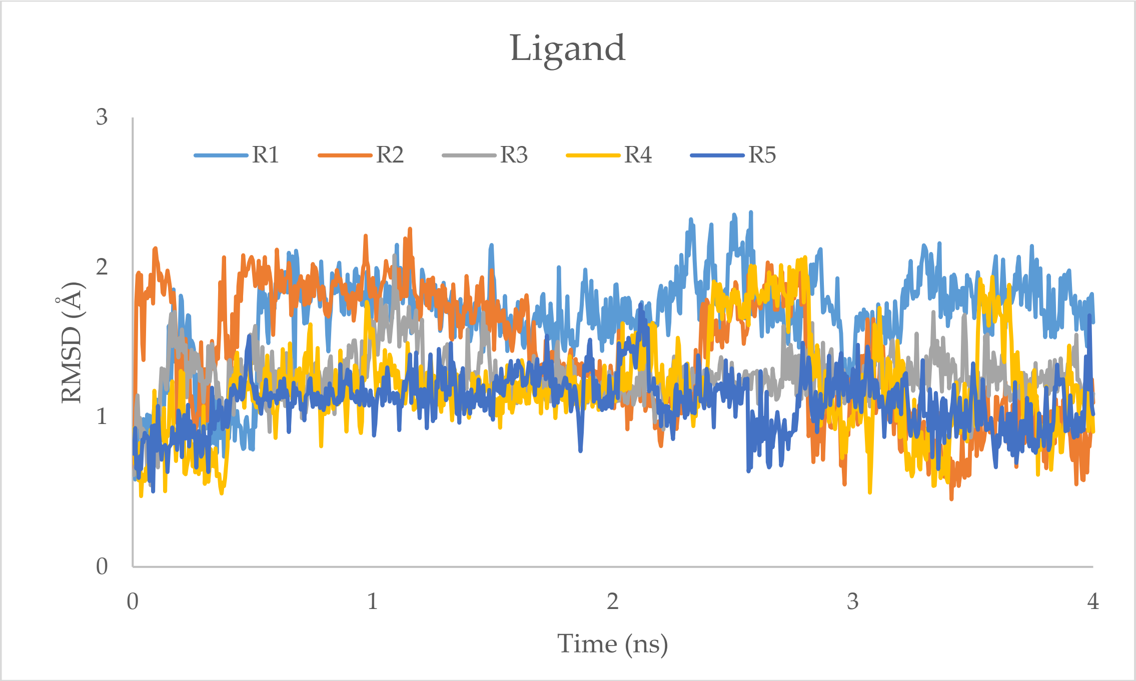

Figure S8. RMSD plots for the complex with AKR. Separate plots are presented for the protein backbone (top) and VATR131 (bottom). Each MD replica is labelled as R1 to R5.

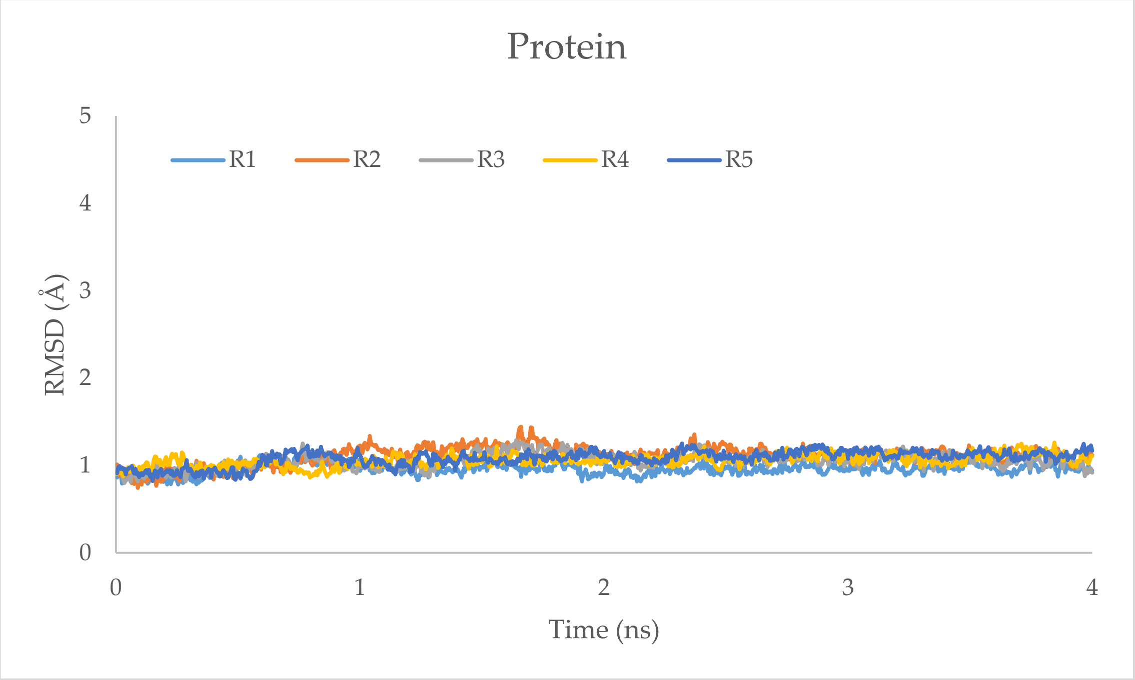

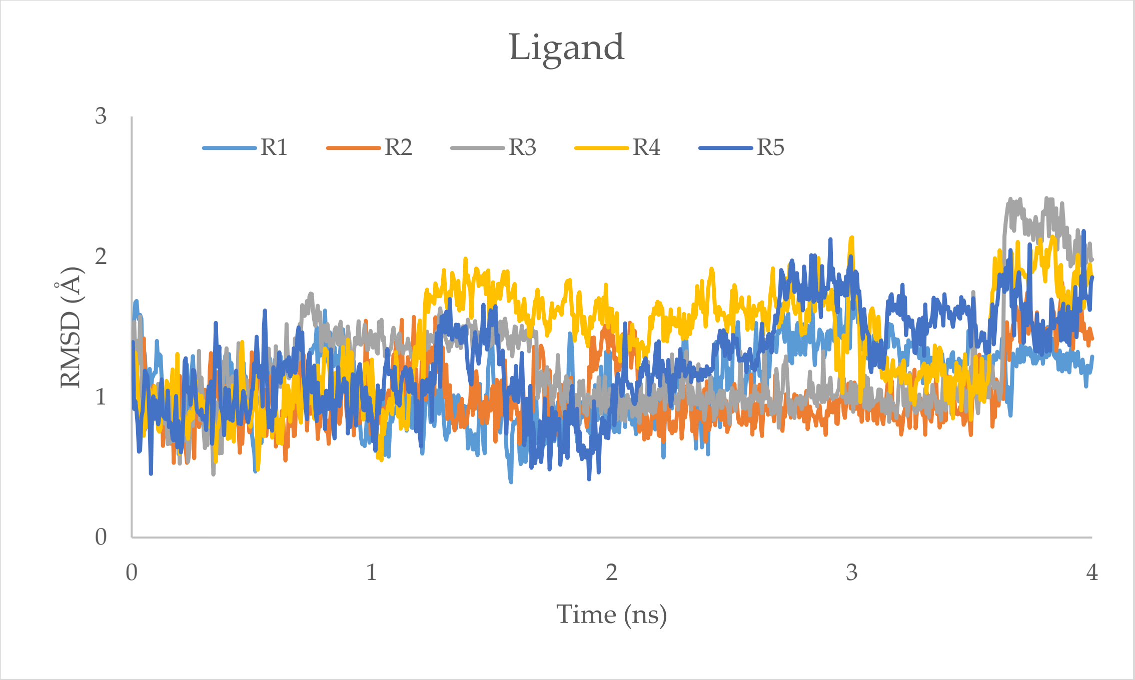

Figure S9. RMSD plots for the complex with HSP60-1. Separate plots are presented for the protein backbone (top) and VATR131 (bottom). Each MD replica is labelled as R1 to R5.

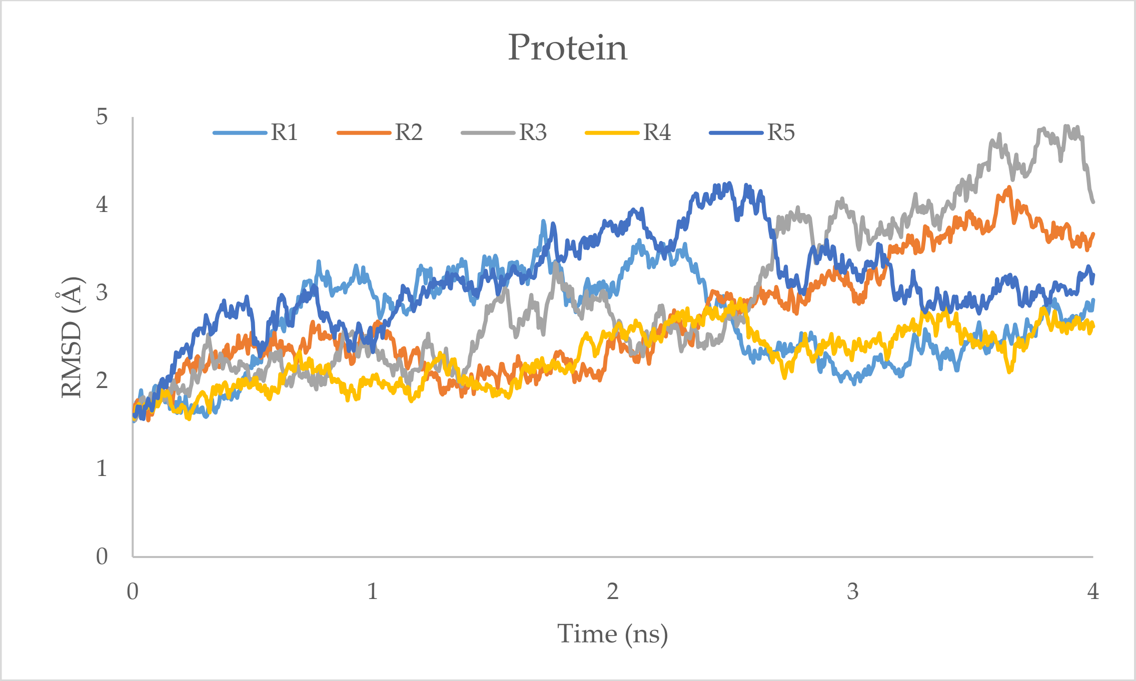

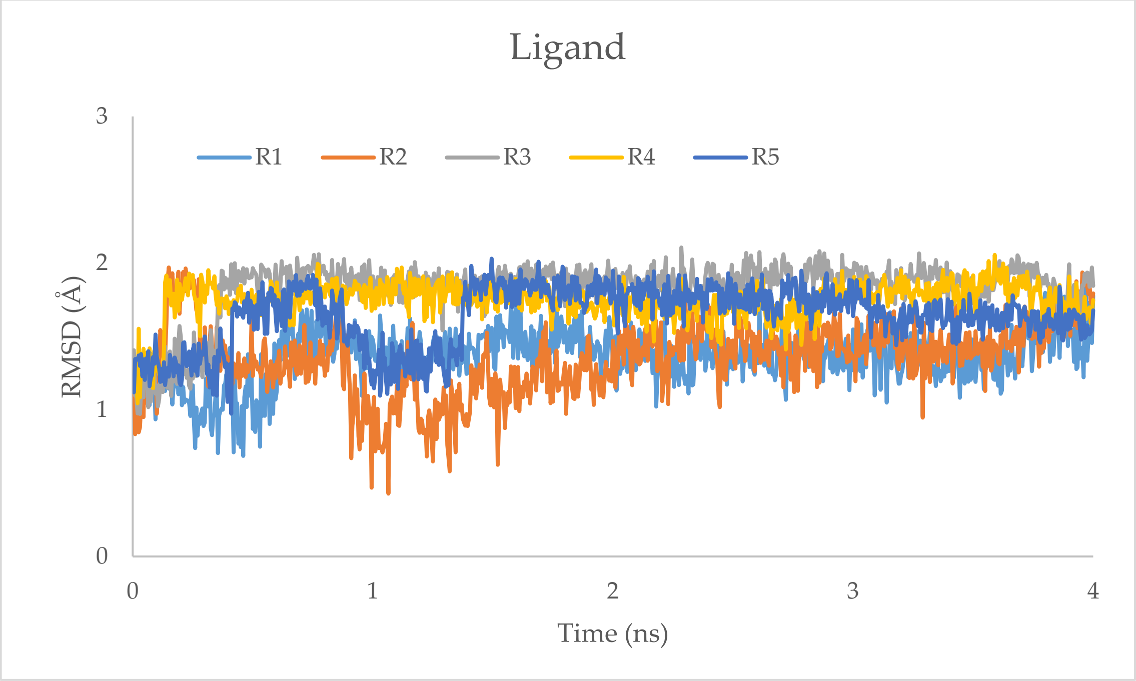

Figure S10. RMSD plots for the complex with PKA. Separate plots are presented for the protein backbone (top) and VATR131 (bottom). Each MD replica is labelled as R1 to R5.

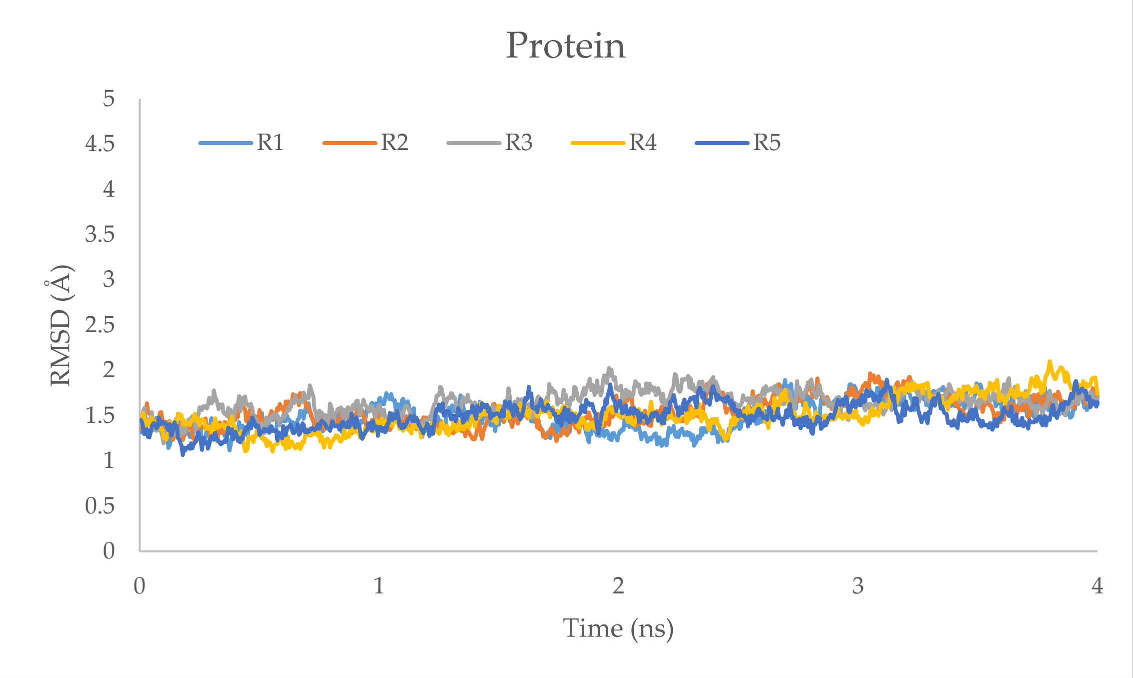

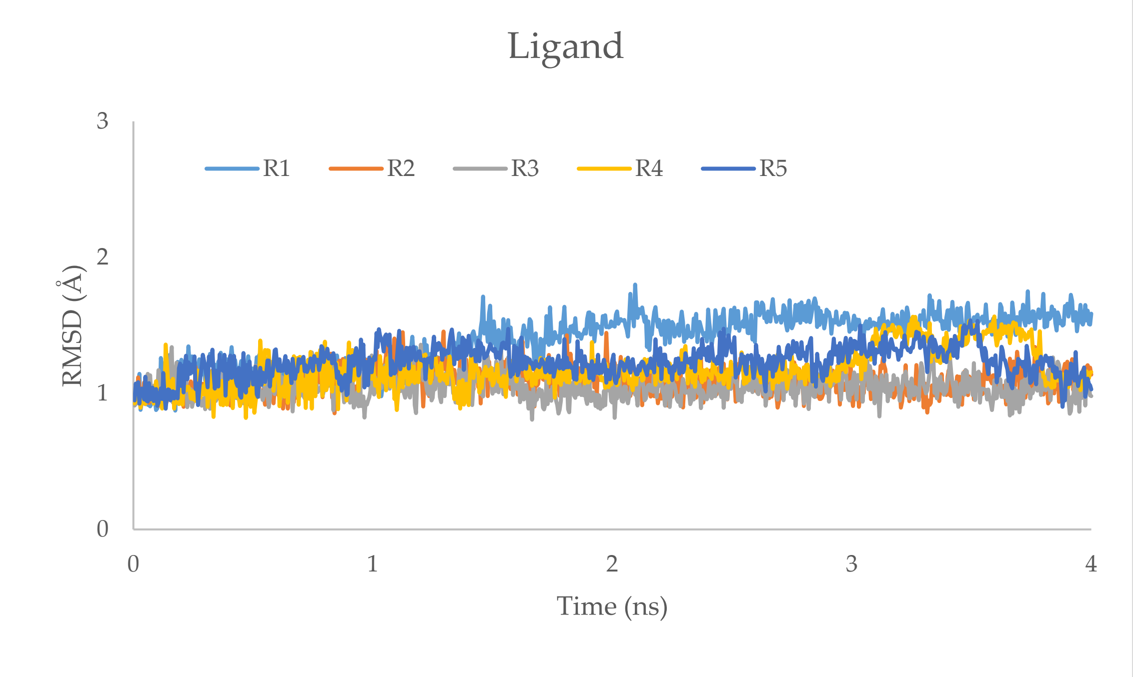

Figure S11. RMSD plots for the complex with HSP60-2. Separate plots are presented for the protein backbone (top) and VATR131 (bottom). Each MD replica is labelled as R1 to R5.

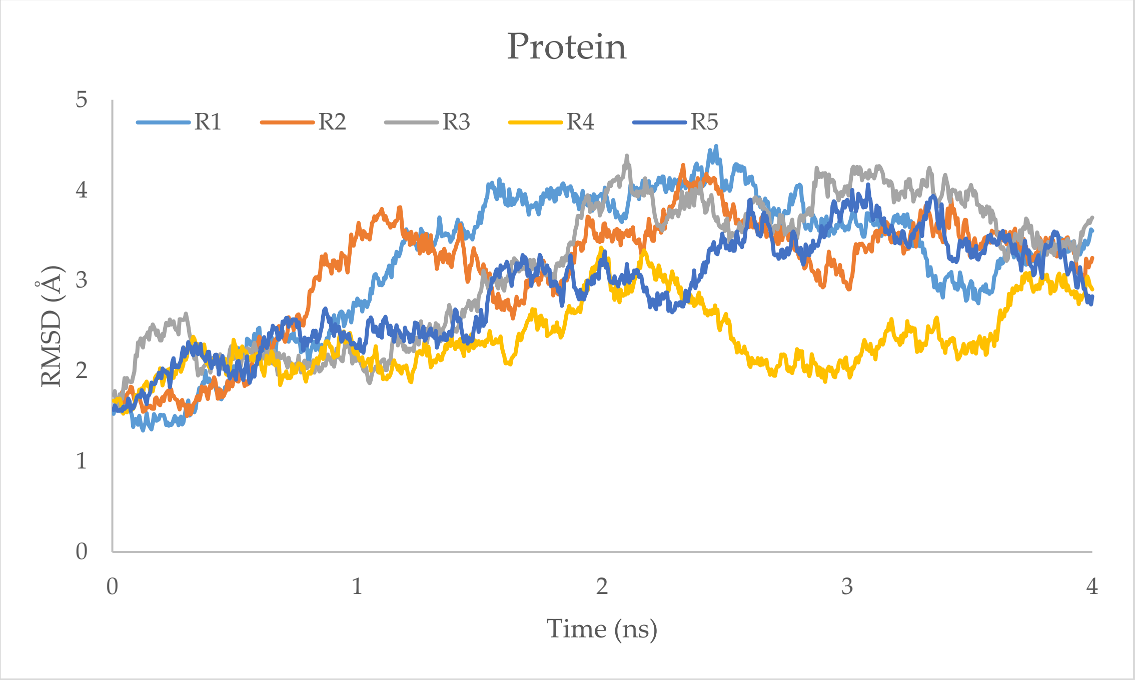

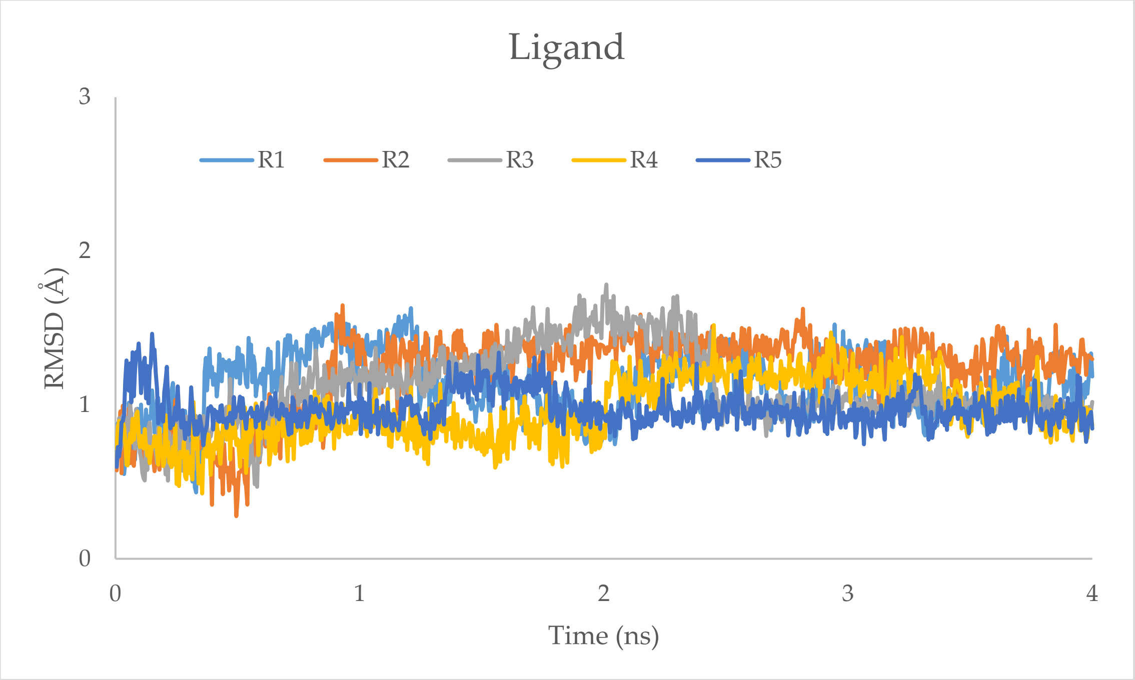

Figure S12. RMSD plots for the complex with HSP60-3. Separate plots are presented for the protein backbone (top) and VATR131 (bottom). Each MD replica is labelled as R1 to R5.

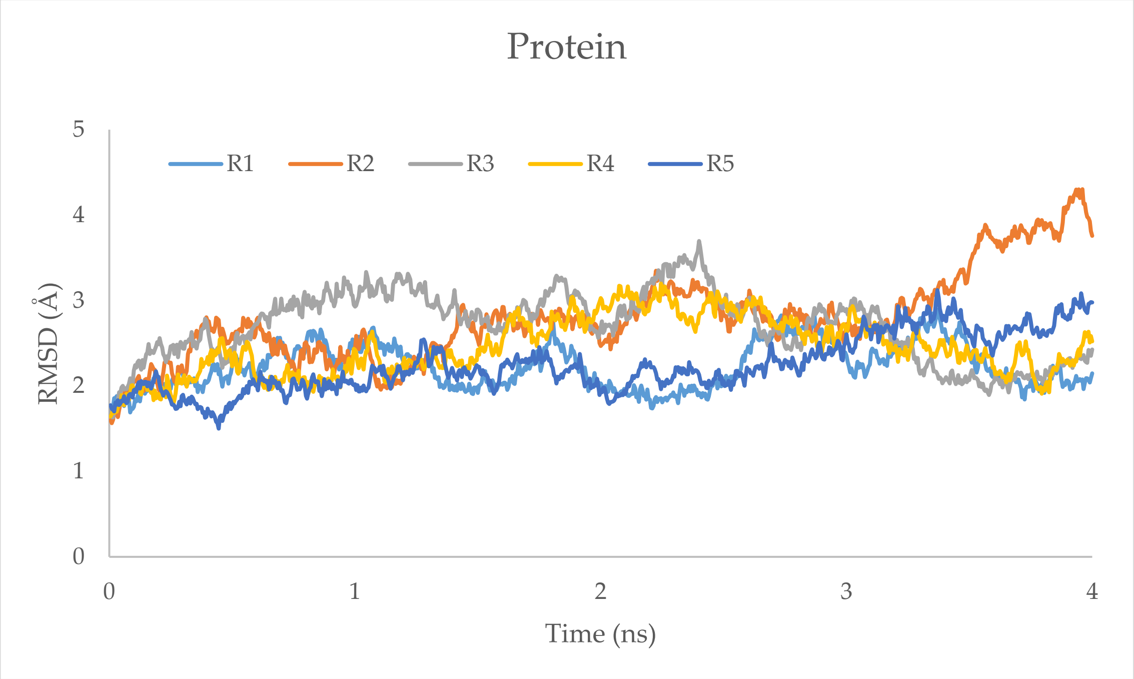

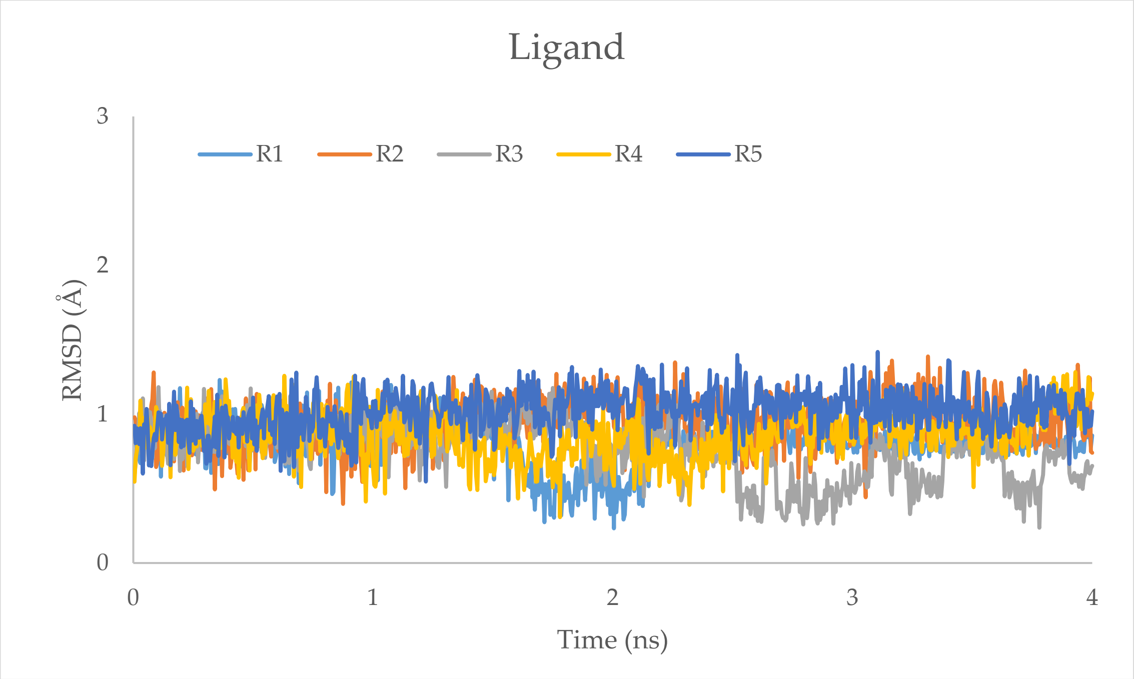

Figure S13. Flexibility of protein backbone in the five MD replicas (R1 to R5) for CPB. The color scale goes from less flexible (blue) to highly flexible (red)

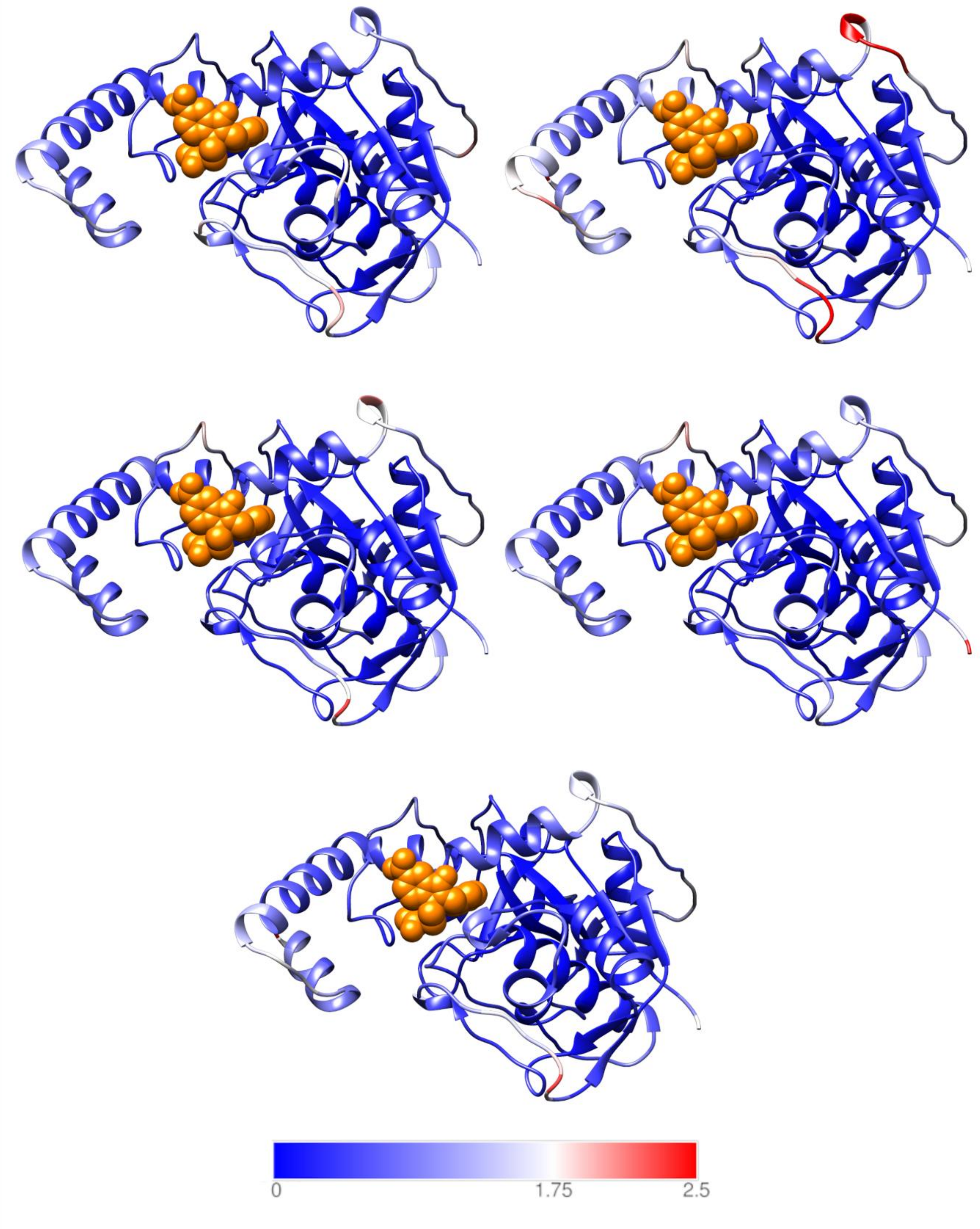

Figure S14. Flexibility of protein backbone in the five MD replicas (R1 to R5) for PKAC1. The color scale goes from less flexible (blue) to highly flexible (red)

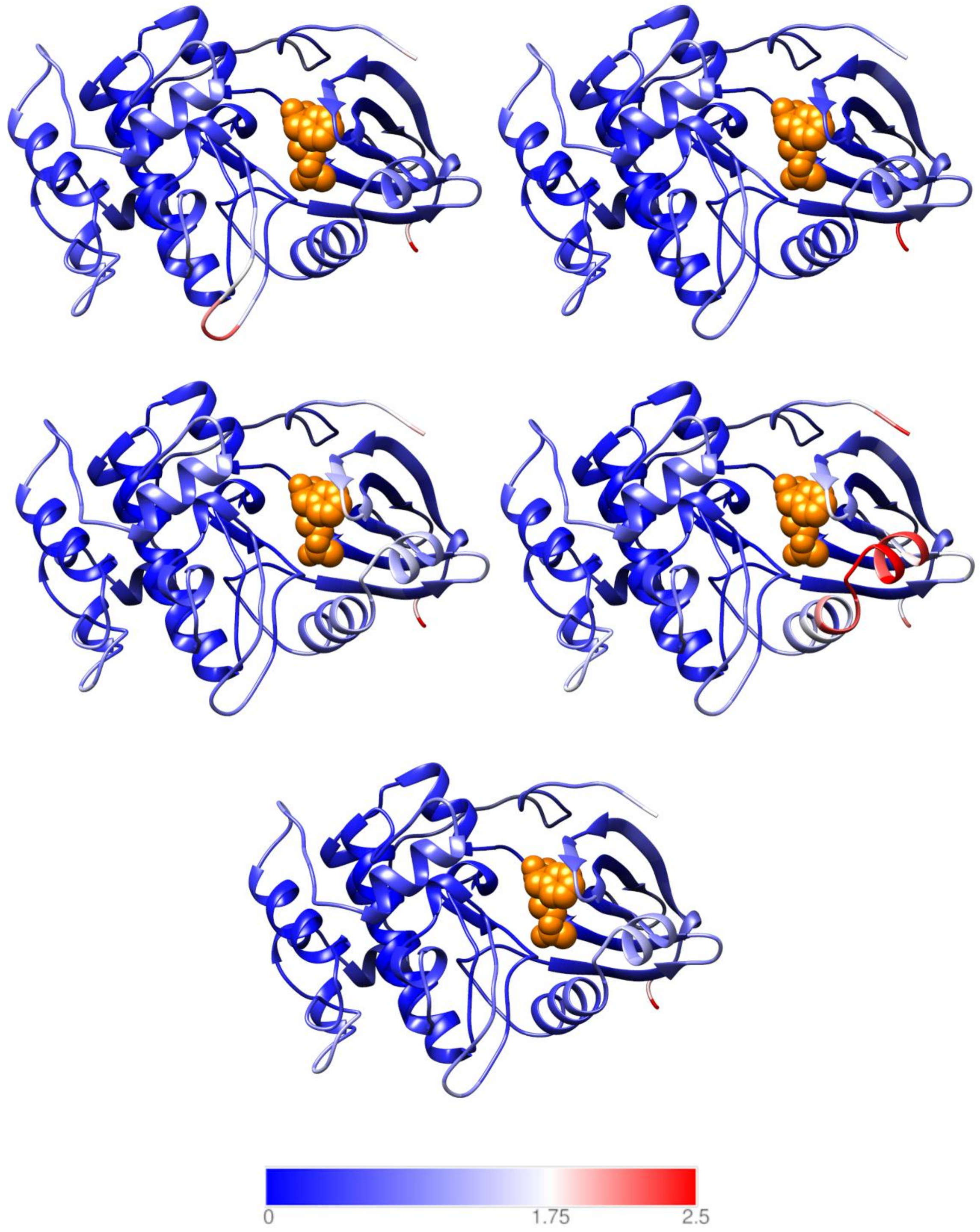

Figure S15. Flexibility of protein backbone in the five MD replicas (R1 to R5) for CPA. The color scale goes from less flexible (blue) to highly flexible (red)

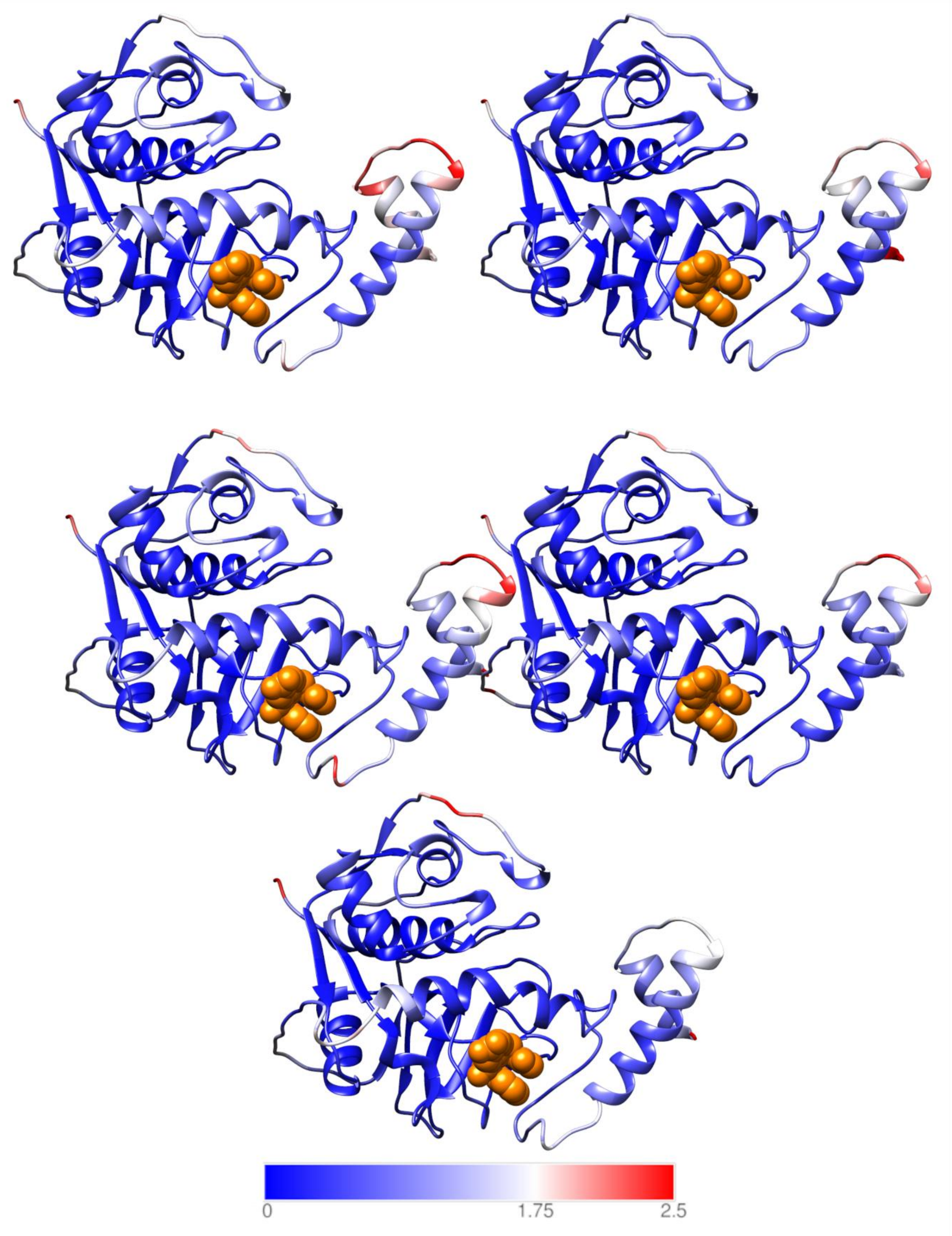

Figure S16. Flexibility of protein backbone in the five MD replicas (R1 to R5) for HSP60. The color scale goes from less flexible (blue) to highly flexible (red)

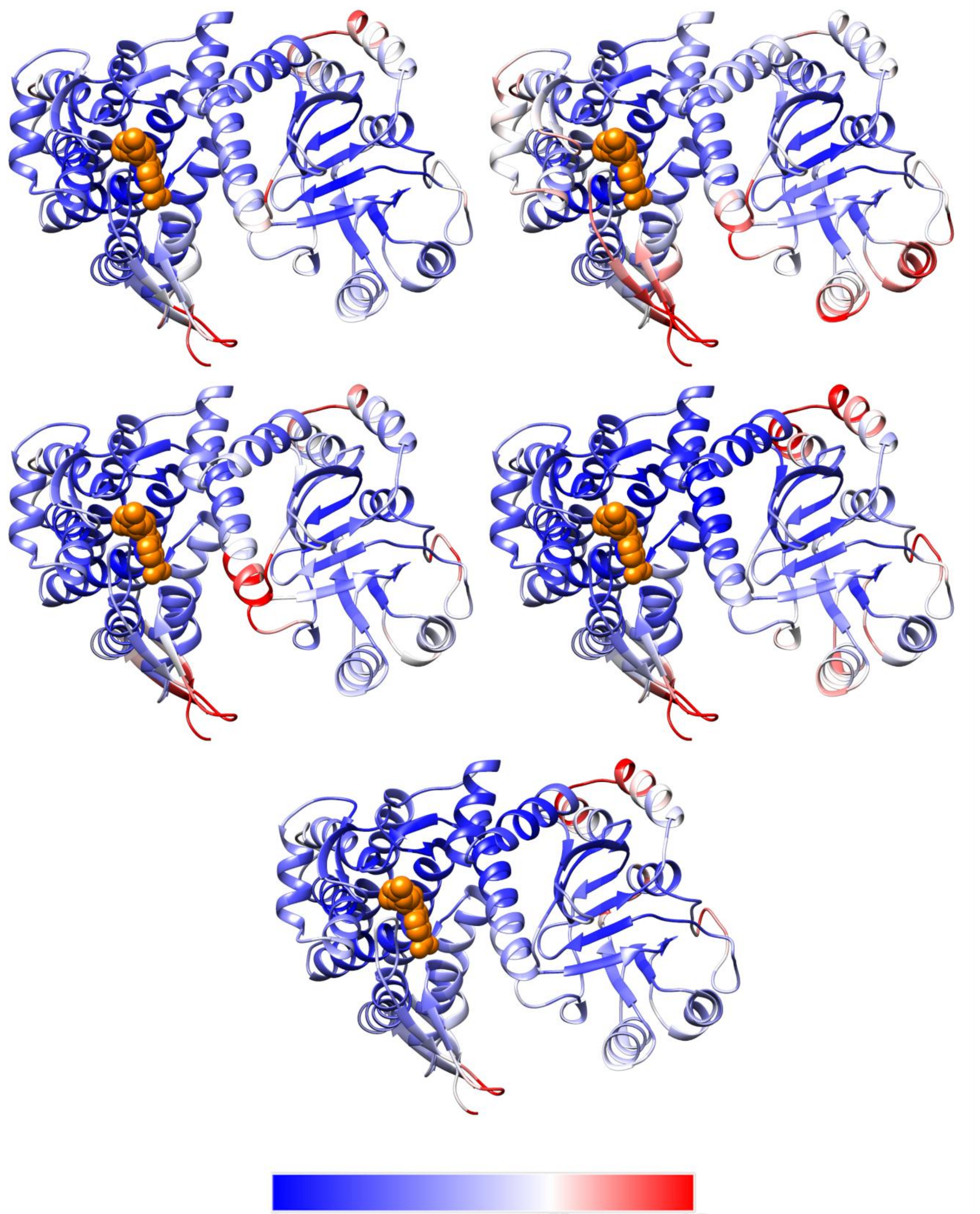

Figure S17. Flexibility of protein backbone in the five MD replicas (R1 to R5) for PGFS. The color scale goes from less flexible (blue) to highly flexible (red)

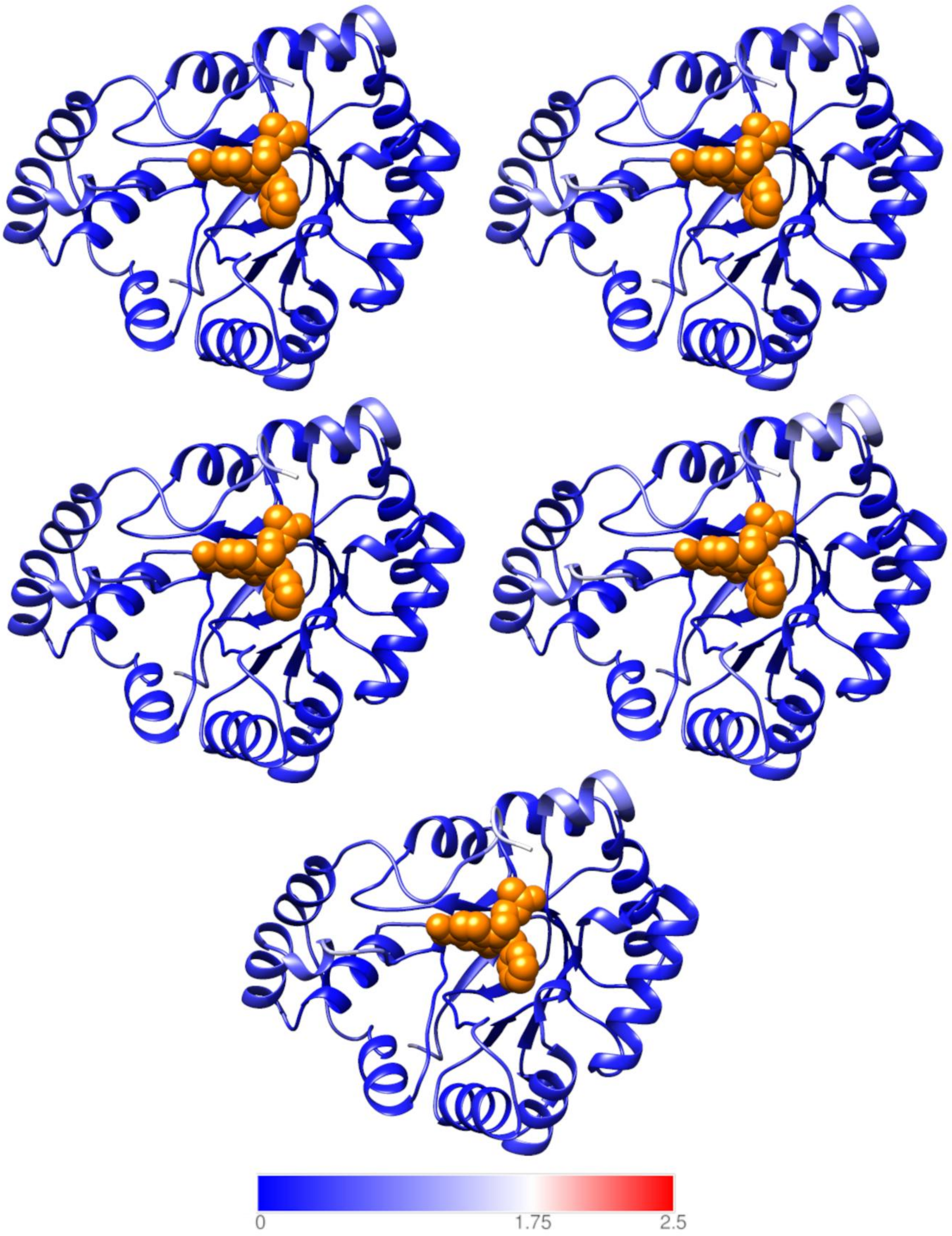

Figure S18. Flexibility of protein backbone in the five MD replicas (R1 to R5) for PGFS2. The color scale goes from less flexible (blue) to highly flexible (red)

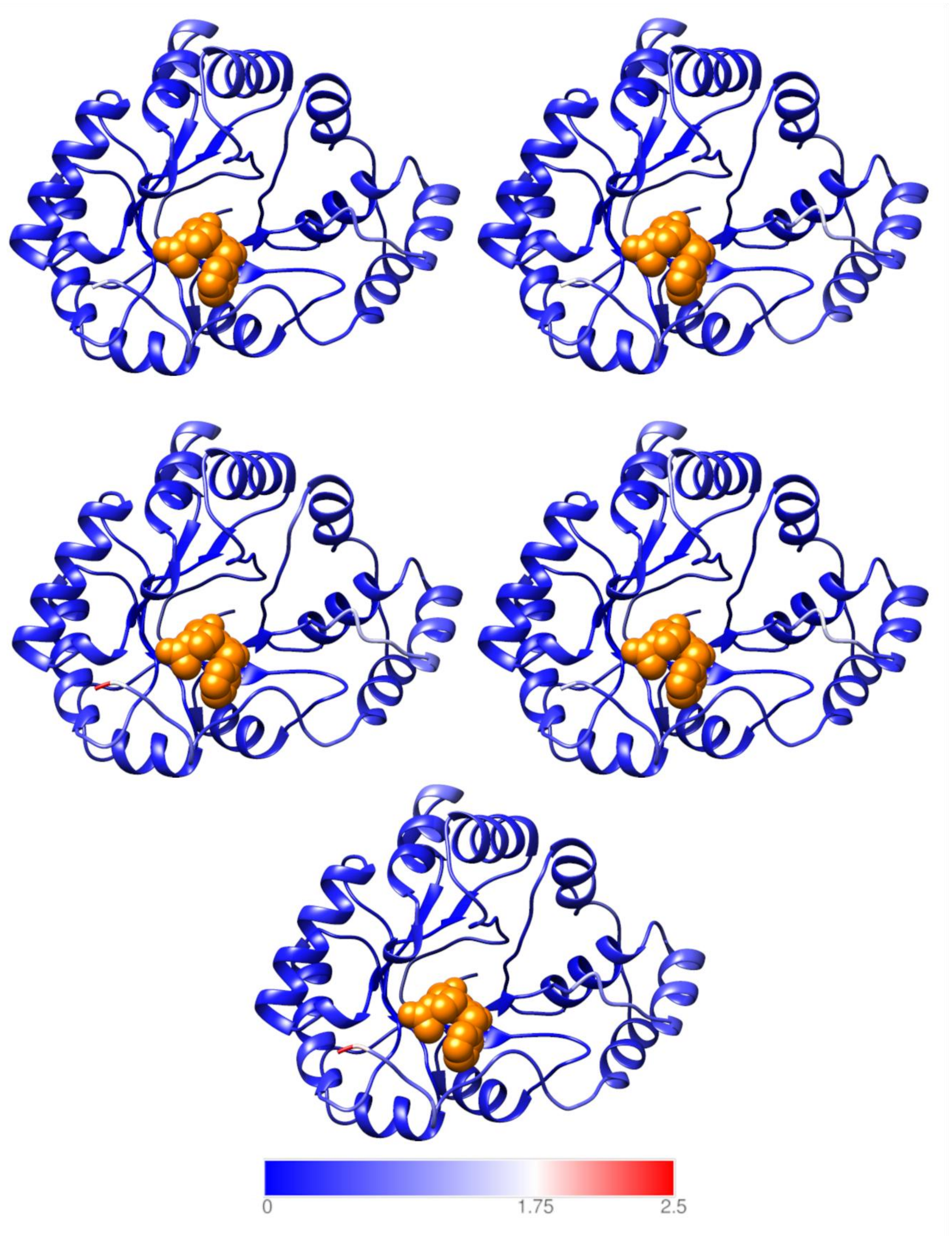

Figure S19. Flexibility of protein backbone in the five MD replicas (R1 to R5) for AKR. The color scale goes from less flexible (blue) to highly flexible (red)

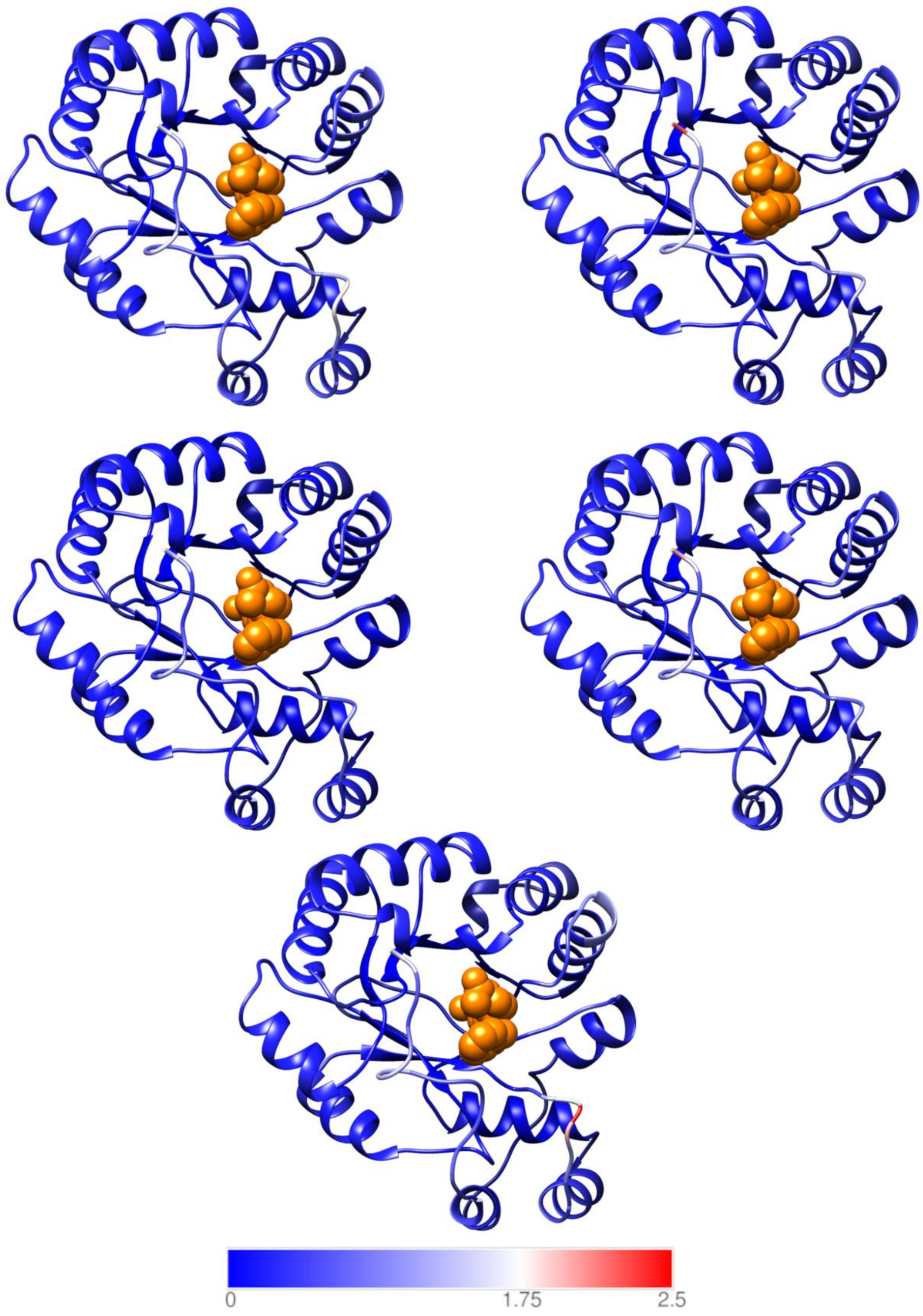

Figure S20. Flexibility of protein backbone in the five MD replicas (R1 to R5) for HSP60-1. The color scale goes from less flexible (blue) to highly flexible (red)

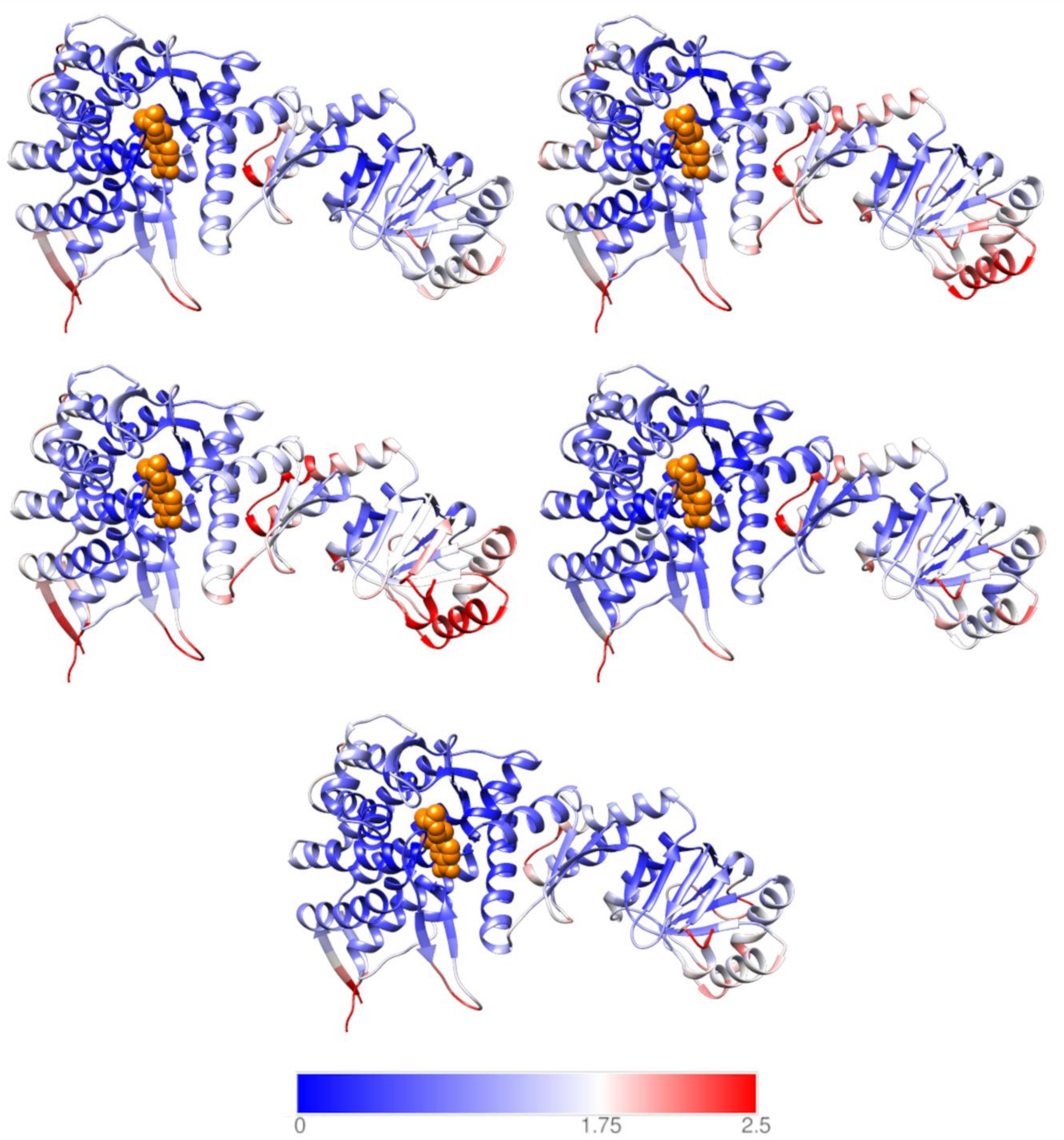

Figure S21. Flexibility of protein backbone in the five MD replicas (R1 to R5) for PKA. The color scale goes from less flexible (blue) to highly flexible (red)

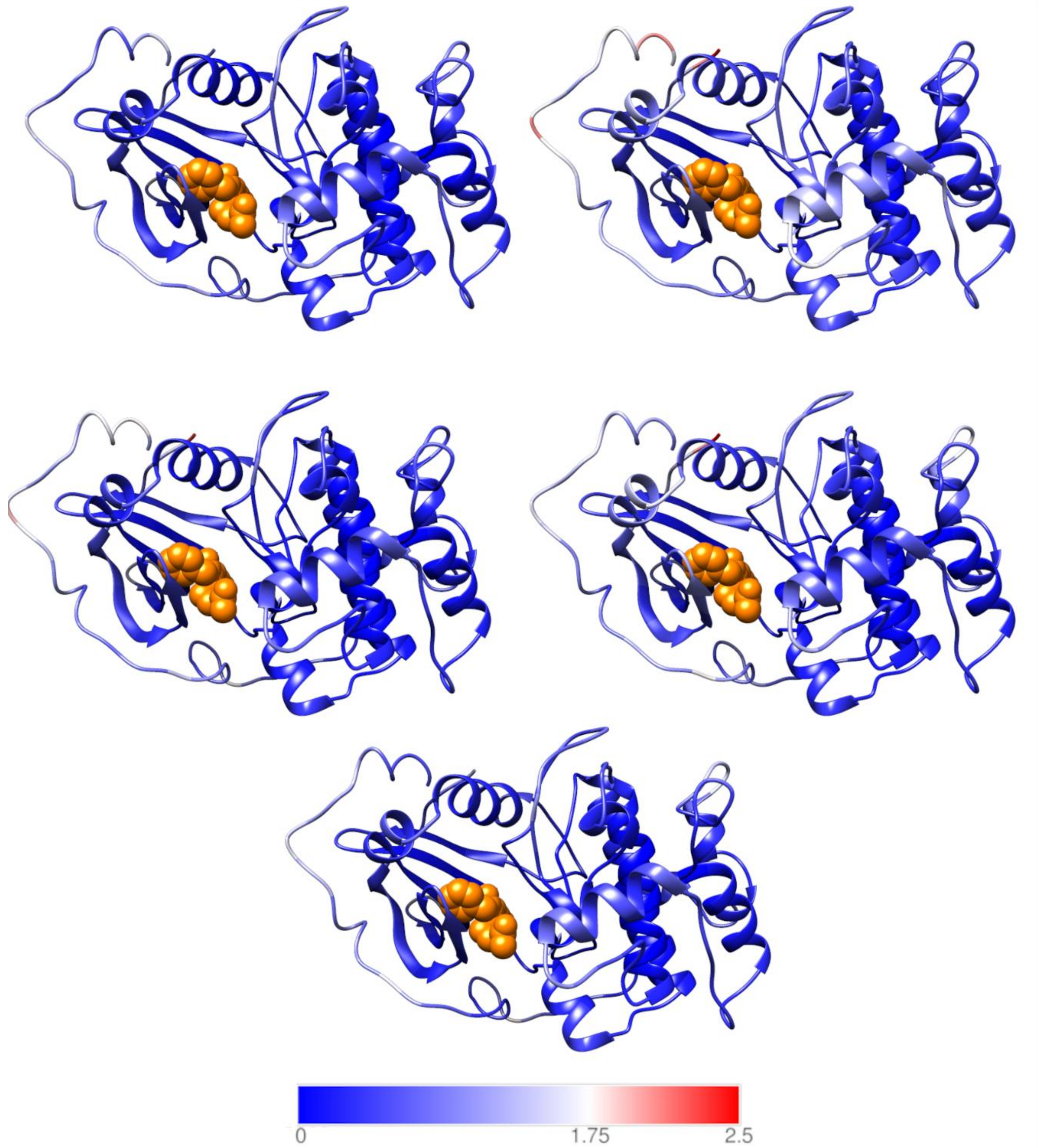

Figure S22. Flexibility of protein backbone in the five MD replicas (R1 to R5) for HSP60-2. The color scale goes from less flexible (blue) to highly flexible (red)

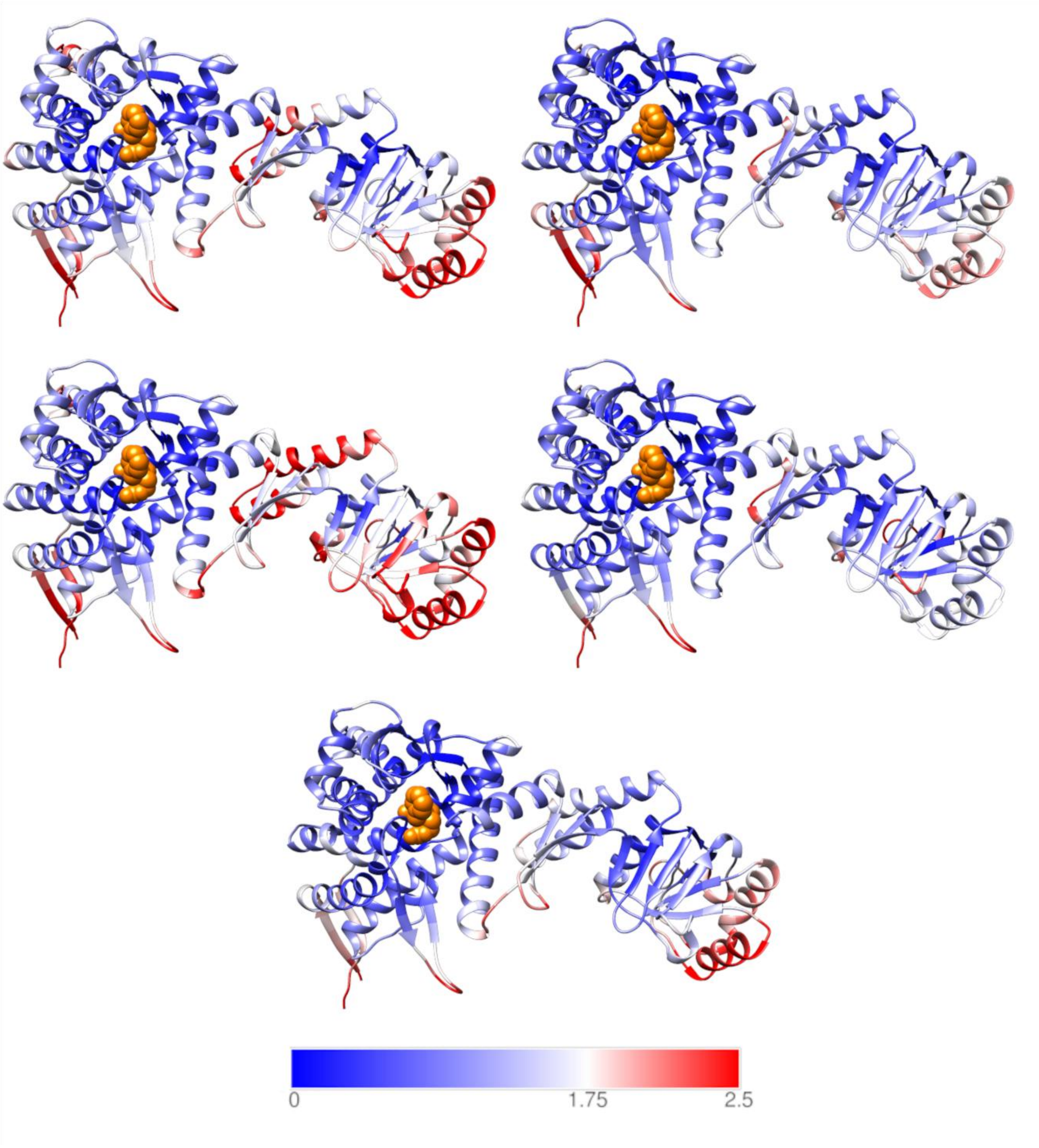

Figure S23. Flexibility of protein backbone in the five MD replicas (R1 to R5) for HSP60-3. The color scale goes from less flexible (blue) to highly flexible (red)

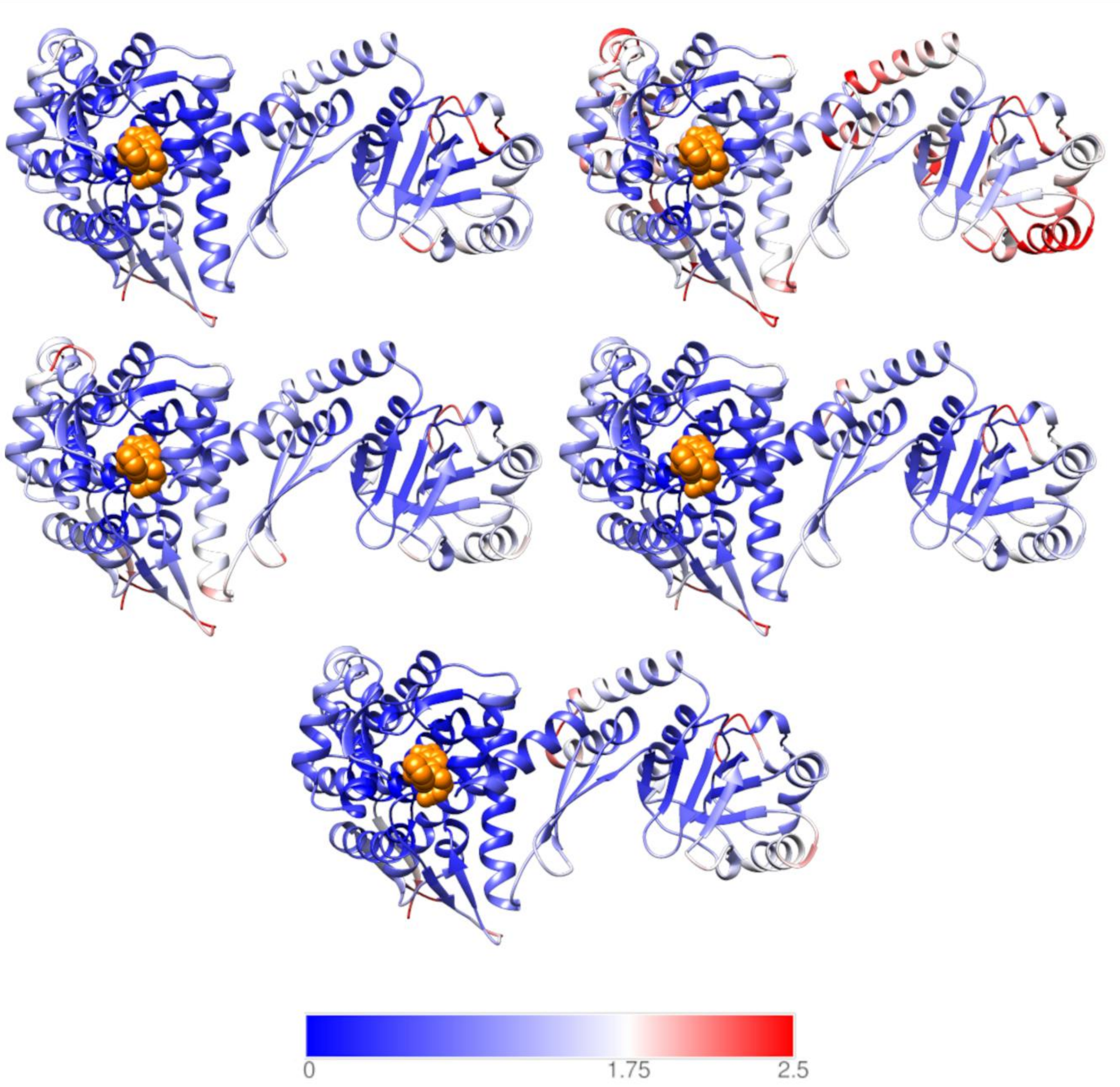

Supplement: Supplementary file 1 [file pharmaceuticals-18-01489-s001.zip › pharmaceuticals-3845985-supplementary.pdf]
